# Long-Term Effects of Early-Life Antibiotic Exposure on Resistance to Subsequent Bacterial Infection

**DOI:** 10.1128/mBio.02820-19

**Published:** 2019-12-24

**Authors:** Claire Roubaud-Baudron, Victoria E. Ruiz, Alexander M. Swan, Bruce A. Vallance, Ceren Ozkul, Zhiheng Pei, Jackie Li, Thomas W. Battaglia, Guillermo I. Perez-Perez, Martin J. Blaser

**Affiliations:** a CHU Bordeaux, Pôle de Gérontologie Clinique, Bordeaux, France; b University of Bordeaux, INSERM, UMR1053 Bordeaux Research in Translational Oncology, BaRITOn, Bordeaux, France; c Department of Medicine, New York University Langone Medical Center, New York, New York, USA; d Department of Biology, St. Francis College, Brooklyn, New York, USA; e Division of Gastroenterology, Department of Pediatrics, University of British Columbia, Vancouver, British Columbia, Canada; f Department of Pharmaceutical Microbiology, Hacettepe University School of Pharmacy, Ankara, Turkey; g Department of Pathology, New York University Langone Medical Center, New York, New York, USA; h Center for Advanced Biotechnology and Medicine, Rutgers University, New Brunswick, NJ, USA; Institut Pasteur

**Keywords:** *Citrobacter rodentium*, pathogen-induced colitis, gastrointestinal microbiota, host resistance, murine model, bioluminescence, colonic inflammation, antibiotics

## Abstract

Early-life antibiotic exposure may provoke long-lasting microbiota perturbation. Since a healthy gut microbiota confers resistance to enteric pathogens, we hypothesized that early-life antibiotic exposure would worsen the effects of a bacterial infection encountered as an adult. To test this hypothesis, C57BL/6 mice received a 5-day course of tylosin (macrolide), amoxicillin (β-lactam), or neither (control) early in life and were challenged with Citrobacter rodentium up to 80 days thereafter. The early-life antibiotic course led to persistent alterations in the intestinal microbiota and even with pathogen challenge 80 days later worsened the subsequent colitis. Compared to exposure to amoxicillin, exposure to tylosin led to greater disease severity and microbiota perturbation. Transferring the antibiotic-perturbed microbiota to germfree animals led to worsened colitis, indicating that the perturbed microbiota was sufficient for the increased disease susceptibility. These experiments highlight the long-term effects of early-life antibiotic exposure on susceptibility to acquired pathogens.

## Abstract (precis)

The gastrointestinal microbiota protects hosts from enteric infections; while antibiotics, by altering the microbiota, may diminish this protection. We show that after early-life exposure to antibiotics host susceptibility to enhanced Citrobacter rodentium-induced colitis is persistent and that this enhanced disease susceptibility is transferable by the antibiotic-altered microbiota. These results strongly suggest that early-life antibiotics have long-term consequences on the gut microbiota and enteropathogen infection susceptibility.

## INTRODUCTION

The gastrointestinal tract carries highly complex and dense microbial populations (1–3), known to have important beneficial functions, including nutriment absorption, vitamin synthesis, and protection against pathogens (4). The microbiota protects the host from pathogen invasion either directly, by producing antimicrobial substances or competing for nutrients or space, or indirectly, by eliciting mucosal immune responses (5–7).

Antibiotics affect gut microbiota composition (8, 9). Studies since the 1950s have shown that exposure of mice to antibiotics prior to experimental inoculation with *Salmonella* worsens outcome (10, 11). In humans, antibiotic exposure enhances susceptibility to Clostridium difficile infection (12). These observations suggest that antibiotic exposure, by perturbing the composition of the host microbiota, may directly influence the severity of enteric infections. Such model studies have largely been conducted in adult animals in which pathogen exposure follows closely after the antibiotic course has ended (7, 13, 14).

Sharing host-interactive mechanisms with certain pathogenic strains of Escherichia coli (15), Citrobacter rodentium is a mouse-restricted Gram-negative bacterium that induces colitis (16, 17). The severity and consequences of C. rodentium infection are affected by the host strain genetic background: while NIH or C57BL/6 mice develop diarrhea and weight loss, these ill effects are transient (18) and the mice develop resistance against subsequent infections (19), whereas C3H-HeJ mice suffer high mortality (20). Interstrain variation in host microbiota composition also influences the outcome of C. rodentium infection (14, 21–23). That transferring host microbiota from resistant to susceptible mouse strains or vice versa affects mortality (21, 23, 24) indicates that the composition of the host microbiota affects disease outcome. In particular, specific commensals, e.g., segmented filamentous bacteria (“*Candidatus* Savagella”), augment resistance to C. rodentium (22), influencing its colonic colonization (25).Conversely, exposure of adult mice to metronidazole, an antimicrobial with broad antianaerobic activity, led to more severe C. rodentium infection (14).

In both mice (26) and humans (27), early life is the crucial period for the development of the mature adult microbiome. Consequently, exposure to antibiotics early in life may induce long-term alterations in the diversity, composition, and metagenomic content of the microbiota (28), even following a single antibiotic course (29, 30), and the antibiotic-induced selection of the microbiota was both necessary and sufficient for changing immunological development (30).

All over the world, young children are receiving multiple antibiotic courses, often inappropriately for mild and self-limiting conditions (31). Although epidemiologic studies provide evidence that such exposures during early life may increase susceptibility to subsequent infections (32, 33), this hypothesis has not yet been directly tested experimentally. Therefore, we examined the effects of a single early-life antibiotic course on the characteristics of subsequent pathogen challenge. We found that early-life antibiotic exposure worsened the outcome of C. rodentium infection in adult life and that the perturbed microbiota was sufficient for transferring the enhanced effect to antibiotic-naive recipient mice.

## RESULTS

### Exposure to tylosin accelerates the course of C. rodentium infection when mice are challenged 1 day after stopping the antibiotic.

**FIG 1:**
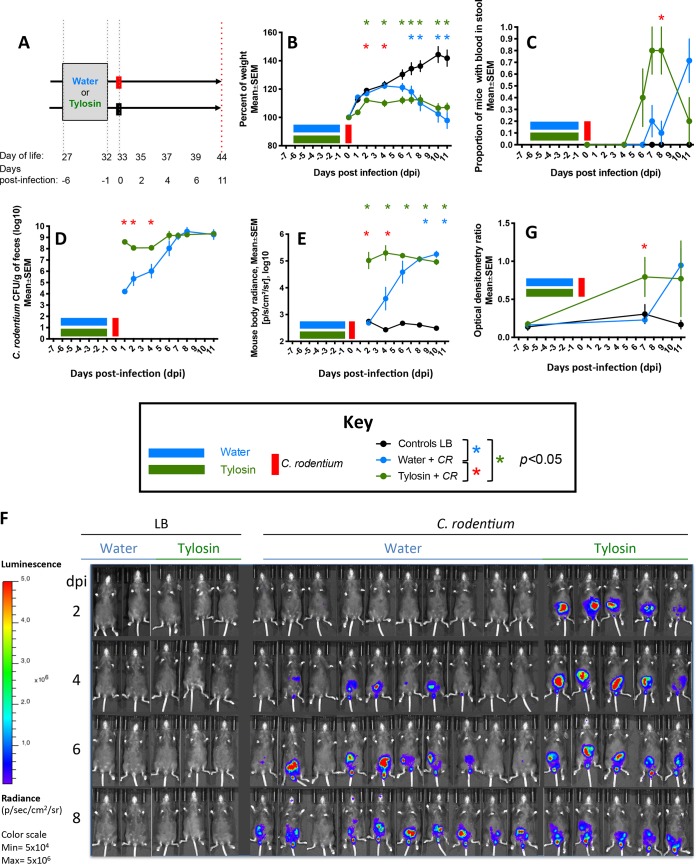
Characteristics of mice challenged with C. rodentium (or Luria broth) 1 day after stopping tylosin or water. (A) Study design. Twenty-one-day-old mice received a 5-day course of tylosin (*n* = 8) or water (*n* = 12). The following day, mice from both treatment groups were challenged by *luxCDABE* C. rodentium (*CR*) (water + *CR* [*n* = 10; blue] or tylosin + *CR* [*n* = 5; green]) or Luria broth (LB) (water + LB [*n* = 2] or tylosin + LB [*n* = 3]; black). The group treated with water plus *CR* consisted of 5 mice each that received one or two *CR* gavages (see Fig. S1A to D). Fecal pellets were collected before and after antibiotic exposure and challenge, and bioluminescence was imaged every 2 days after infection. All mice were sacrificed 11 days postinfection. (B) Body weight over time, calculated as a percentage of prechallenge weight. (C) Proportion of mice with fecal occult blood. (D) Quantitation of C. rodentium in fecal culture. (E) Quantitation of C. rodentium by *in vivo* bioluminescent imaging. (F) *In vivo* bioluminescent images of mouse bodies. (G) Anti-C. rodentium IgM antibody levels.

To determine whether antibiotic exposure can affect host resistance even after it is no longer being administered, we challenged young adult mice with C. rodentium immediately after a 5-day tylosin (macrolide) course was completed; control mice were unexposed to tylosin and/or inoculated with Luria broth (LB) rather than C. rodentium (Fig. 1A). All uninfected mice gained weight over time (Fig. 1B; see also Fig. S1E in the supplemental material), whereas all infected mice lost weight (Fig. 1B); however, among infected mice, those that had been exposed to tylosin started losing weight ∼5 days earlier than antibiotic-naive mice. Two infected antibiotic-naive mice died 9 days postinfection (dpi), and all remaining infected mice were sacrificed at 11 dpi due to excessive weight loss. Fecal occult blood was absent in uninfected mice but among infected mice was present ∼1 day earlier and more frequently in those that had been exposed to tylosin than in the antibiotic-naive mice (Fig. 1C). Among infected mice, those that had been tylosin exposed had higher fecal and *in vivo* C. rodentium levels from 1 to 4 dpi than did the antibiotic-naive mice (Fig. 1D to F). Specific IgM antibodies were absent in the sera of mice prior to C. rodentium challenge and were absent at all times in uninfected mice (Fig. 1G). At 7 dpi, specific IgM levels were higher in mice exposed to tylosin than in antibiotic-naive mice (0.80 ± 0.060 versus 0.23 ± 0.012; *P* = 0.015). By the day of sacrifice (11 dpi), IgM levels became similar in both groups of infected mice, whether tylosin exposed or not (Fig. 1G). Specific serum IgG was absent throughout the brief experimental course in all mice (data not shown). This experiment confirmed prior studies indicating that in adult mice, proximate antibiotic exposure worsens subsequent pathogen challenge (14).

10.1128/mBio.02820-19.1 FIG S1 Effects of either C. rodentium challenge or tylosin exposure (but not both) on host phenotypes (see Fig. 1A). (A to D) Comparison of one or two C. rodentium challenges on host phenotypes. Thirty-three-day-old mice were challenged with C. rodentium twice on consecutive days (*n* = 5) or only once (*n* = 5), and their characteristics were evaluated. (A) Body weight over time, calculated as a percentage of prechallenge weight. (B) Proportion of mice with fecal occult blood. (C) Quantitation of C. rodentium in fecal culture. (D) Anti-C. rodentium IgM antibody levels. (E) Host characteristics of mice exposed to tylosin or not, without subsequent C. rodentium challenge. Twenty-seven-day-old mice were given tylosin (*n* = 3) or not (*n* = 2), for 5 days, and their characteristics were observed. (Left axis) Body weight over time, calculated as a percentage of prechallenge weight (solid lines). (Right axis) Anti-C. rodentium IgM antibody levels (dashed lines). For symbols, see key. Download FIG S1, PDF file, 11.5 MB. Copyright © 2019 Roubaud-Baudron et al. 2019 Roubaud-Baudron et al. This content is distributed under the terms of the Creative Commons Attribution 4.0 International license.

### Effects of tylosin exposure and subsequent C. rodentium challenge on microbiota.

**FIG 2:**
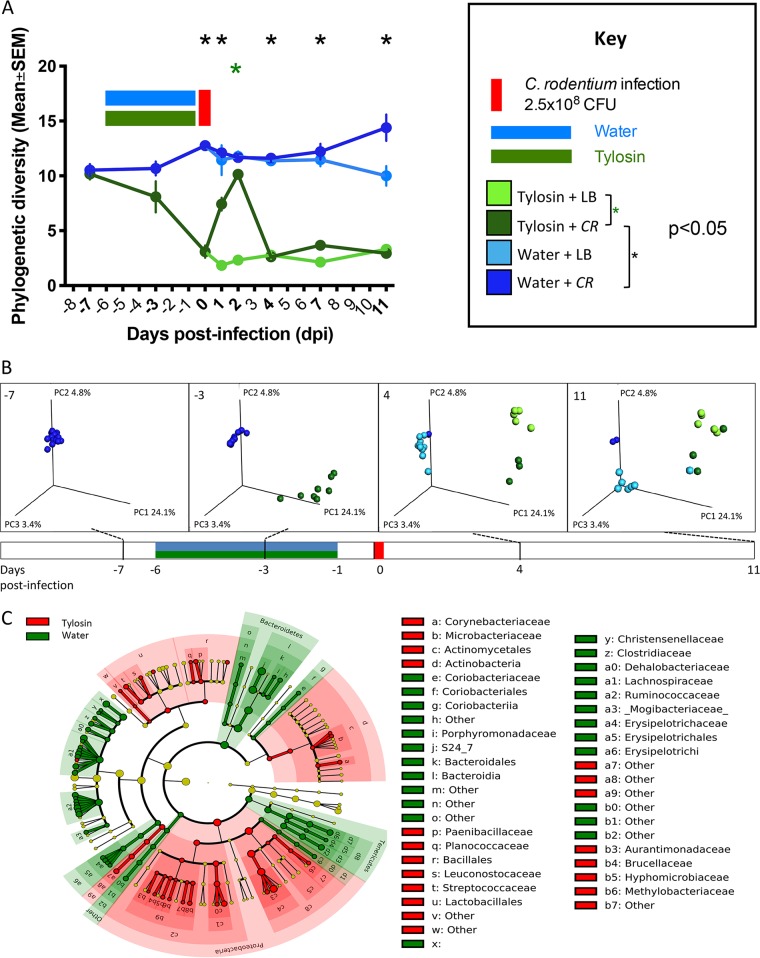
Gut microbiota characteristics of mice challenged with C. rodentium (or LB) 1 day after stopping tylosin (or water). For the study design, see the legend to Fig. 1A. (A) α-diversity in fecal samples. Using the phylogenetic diversity (PD whole tree) metric, α-diversity was measured in fecal samples obtained at the times pre- and postinfection shown in bold. Mice were exposed to tylosin (or water) and then challenged with *CR* (or LB). (B) Unweighted UniFrac analysis of fecal specimens from four time points visualized by principal-coordinate analysis (PCoA) (for statistical significance, see Table S1). The three components explain 32.3% of the total variance, and the colors are as in panel A. (C) LefSe analysis, with cladogram showing significantly differential taxa between tylosin- and water-exposed mice at day 0, immediately prior to infection. Shading indicates significant overrepresentation of the indicated taxa (*P* < 0.05; LDA [linear discriminant analysis] > 2).

In the model system with adult mice, we next sought to assess how substantially the tylosin exposure affected the gastrointestinal microbiota. Tylosin exposure significantly decreased community richness (Fig. 2A) and affected community structure (β-diversity) (Fig. 2B), as we now expected (28, 30). Tylosin exposure decreased the relative abundances of certain anaerobes (e.g., *Bacteroidetes* and *Tenericutes*) and increased *Proteobacteria* (Fig. 2C). Among tylosin-exposed mice, C. rodentium challenge transiently (2 dpi) increased α-diversity (*P* < 0.001) (Fig. 2A). The C. rodentium challenge further affected β-diversity, but these effects appeared earlier (4 dpi) in the tylosin-exposed mice (Fig. 2B). This experiment indicates that in adult mice, both the tylosin exposure and C. rodentium challenge affect microbiota community richness and structure, with effects persisting until the time of sacrifice in this acute infection model.

10.1128/mBio.02820-19.4 TABLE S1 Summary of Adonis and analysis of similarity (ANOSIM) testing of unweighted UniFrac distances, related to Fig. 2B. Download Table S1, DOCX file, 0.01 MB. Copyright © 2019 Roubaud-Baudron et al. 2019 Roubaud-Baudron et al. This content is distributed under the terms of the Creative Commons Attribution 4.0 International license.

### Mice exposed to early-life antibiotics develop enhanced colitis when challenged 23 days later with C. rodentium.

**FIG 3:**
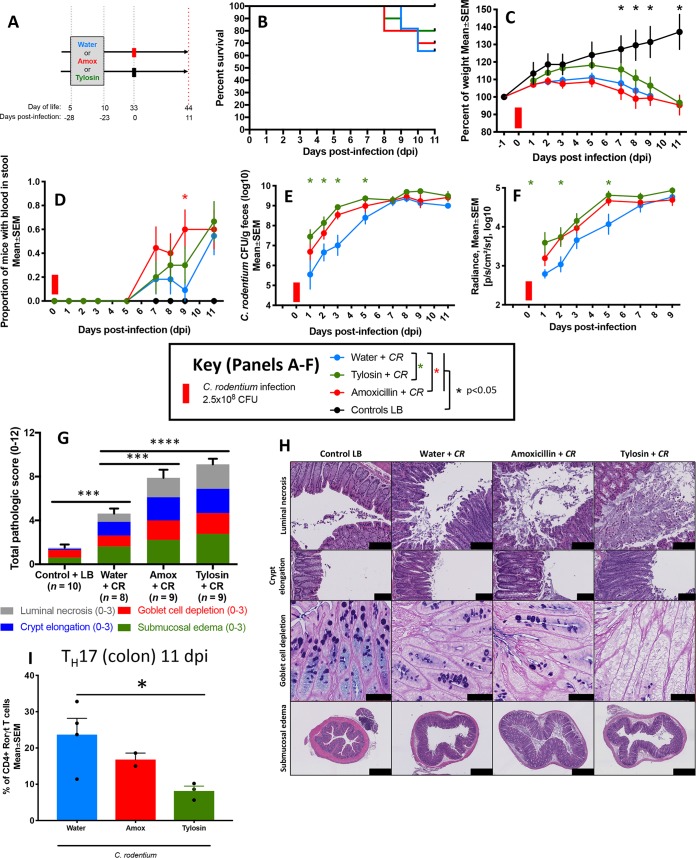
Characteristics of mice challenged with C. rodentium (or LB) 23 days after antibiotic or water exposure. (A) Study design. Five-day-old pups received a 5-day course of tylosin (*n* = 13), amoxicillin (*n* = 13), or water (*n* = 15). Twenty-three days later, pups were challenged with *luxCDABE* C. rodentium (water + *CR* [*n* = 11; blue]), tylosin + *CR* [*n* = 10; green], or amoxicillin + *CR* [*n* = 10; red]) or with LB (as a control) (water + LB [*n* = 4], tylosin + LB [*n* = 3], or amoxicillin + LB [*n* = 3]; black). (B) Percent surviving by exposure group. (C) Body weight over time, calculated as a percentage of prechallenge weight. For the water group, analysis was censored at day 11 due to multiple deaths. (D) Proportion of mice with fecal occult blood. (E) Quantitation of C. rodentium in fecal culture. (F) Quantitation of C. rodentium by *in vivo* bioluminescent imaging. (G) Cumulative histopathology scores (means ± SEM), calculated by evaluating luminal necrosis, crypt elongation, goblet cell depletion, and submucosal edema. ***, *P* < 0.01; ****, *P* < 0.0001. (H) Representative H&E-stained (or, in the third row, AB/PAS-stained) distal colonic sections (scale bars: for luminal necrosis, 100 μm; for crypt elongation, 100 μm; for goblet cell depletion, 50 μm; and for submucosal edema, 500 μm). (I) Flow cytometric analysis of colonic CD4^+^ cells after tylosin, amoxicillin, or water exposure and subsequent C. rodentium challenge. Populations were gated on live CD45^+^ CD4^+^ cells, and representative proportions of IL17A^+^ CD4^+^ cells are shown. *, *P* < 0.05. For symbols, see key.

With the development of the experimental system and phenotype measurement in our lab, we now could examine whether early-life antibiotic exposure would influence the course of infection in young adults (33 days of life). In this experiment, nursing dams were exposed to one of two different antibiotics (tylosin or amoxicillin) in their drinking water or not (control) when their pups were 5 to 10 days old. The pups were exposed to therapeutic doses of the antibiotics through their mothers’ milk (28, 30) and at P33 (23 days later) were challenged with C. rodentium or LB (as a control) (Fig. 3A). The antibiotic exposures did not significantly affect immediate survival and weight loss (Fig. 3B and C). However, the early-life-tylosin-exposed mice were more susceptible than the antibiotic-naive mice to C. rodentium invasion immediately following challenge (Fig. 3E and F and Fig. S2A). Amoxicillin exposure worsened colitis severity with increased fecal blood (Fig. 3D). At P44, 34 days after the early-life antibiotic exposure had ceased, the mice exposed to either of the antibiotics had more severe colonic tissue injury from the C. rodentium challenge than did the antibiotic-naive mice (Fig. 3G and H). Among the challenged mice, those exposed to tylosin had a significantly lower proportion of colonic T_H_17 cell levels at sacrifice than did the antibiotic-naive mice; the total number of colonic T_H_17 cells followed the same trend (Fig. 3I and Fig. S3). Among infected mice, there were no significant differences in the frequencies of regulatory (Foxp3^+^) T cells, or T_H_1 (CD4^+^ IFN-γ^+^) cells, between the exposure groups (data not shown).

10.1128/mBio.02820-19.2 FIG S2 Representative *in vivo* bioluminescent images of mice exposed to antibiotics (or water [control]) and subsequently challenged with C. rodentium (or LB [control]). (A) Study design (see the legend to Fig. 3A). (B) Study design (see the legend to Fig. 5A). W, water; A, amoxicillin; T, tylosin. Download FIG S2, PDF file, 11.5 MB. Copyright © 2019 Roubaud-Baudron et al. 2019 Roubaud-Baudron et al. This content is distributed under the terms of the Creative Commons Attribution 4.0 International license.

10.1128/mBio.02820-19.3 FIG S3 Flow cytometric analysis of colonic CD4^+^ cells after tylosin, amoxicillin, or water exposure and subsequent C. rodentium challenge. Populations were gated on live CD45^+^ CD4^+^ cells, and the total number of IL17A^+^ CD4^+^ cells is shown. *, *P* < 0.05. Download FIG S3, PDF file, 0.02 MB. Copyright © 2019 Roubaud-Baudron et al. 2019 Roubaud-Baudron et al. This content is distributed under the terms of the Creative Commons Attribution 4.0 International license.

**FIG 4:**
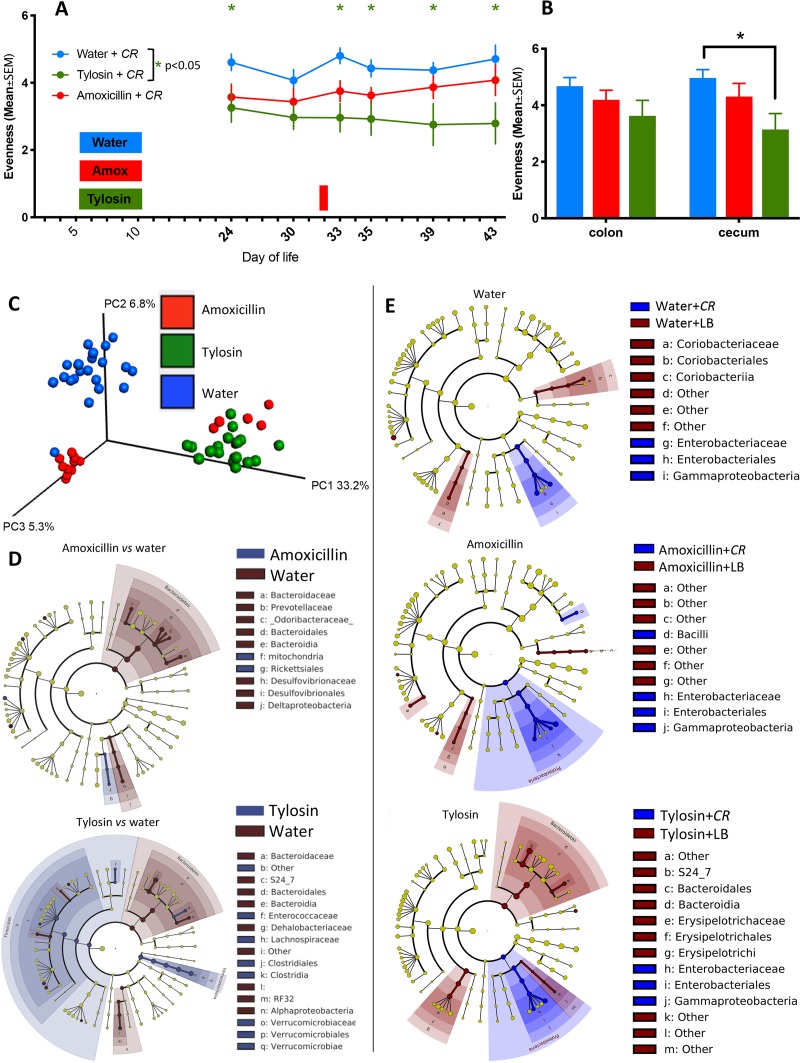
Gut microbiota characteristics of mice challenged with C. rodentium (or LB) 23 days after antibiotic or water exposure. (A) Study design (see the legend to Fig. 3A), with all samples obtained at sacrifice unless as noted. Fecal pellets were collected at the times shown in bold, and α-diversity using the Shannon index of evenness is shown. The red bar on the *x* axis corresponds to C. rodentium challenge. (B) α-Diversity in cecal and colonic samples using Shannon index of evenness. Samples were obtained 11 days after C. rodentium challenge from mice preexposed to water (blue), amoxicillin (red), or tylosin (green). *, *P* < 0.05. (C) Unweighted UniFrac analysis of fecal specimens visualized by PCoA (weaning, 2 days before challenge, and the day of challenge) for each group (for statistical significance, see Table S2). The three components explain 45.3% of the total variance. (D) LefSe analysis, with cladograms showing significantly differential taxa between indicated treatment groups on day 31 (2 days prior to C. rodentium challenge). Shading indicates significant overrepresentation of the indicated taxa (*P* < 0.05; LDA > 2). (E) LefSe analysis between C. rodentium challenged (blue) and unchallenged (brown) mice on day 11 after challenge.

Both prior to and following C. rodentium challenge, early-life tylosin exposure significantly reduced intestinal α-diversity compared to that of antibiotic-naive mice, while amoxicillin exposure had an intermediate effect (Fig. 4A and B). After the early-life exposures, microbial populations remained skewed for at least the next 23 days (Fig. 4C and D) and following C. rodentium challenge (Fig. 4E). Compared to uninfected mice, those challenged with C. rodentium developed higher *Proteobacteria* abundances, as reported previously (34), whether the mice had been exposed to antibiotics or not (Fig. 4E). Among the tylosin-exposed mice, those challenged with C. rodentium had lower abundances of an unidentified *Bacteroidetes* S24-7 family member than those that were uninfected (Fig. 4E). This experiment indicates that 23 days following early-life antibiotic exposure, the host microbiota remained perturbed and that C. rodentium challenge induced more severe infection in these young adult animals that had received either antibiotic in early life.

10.1128/mBio.02820-19.5 TABLE S2 Summary of Adonis and ANOSIM testing of unweighted UniFrac distances, related to Fig. 4C. Download Table S2, DOCX file, 0.01 MB. Copyright © 2019 Roubaud-Baudron et al. 2019 Roubaud-Baudron et al. This content is distributed under the terms of the Creative Commons Attribution 4.0 International license.

### Mice exposed to early-life antibiotics develop enhanced colitis when challenged with C. rodentium as adults, 80 days later.

**FIG 5:**
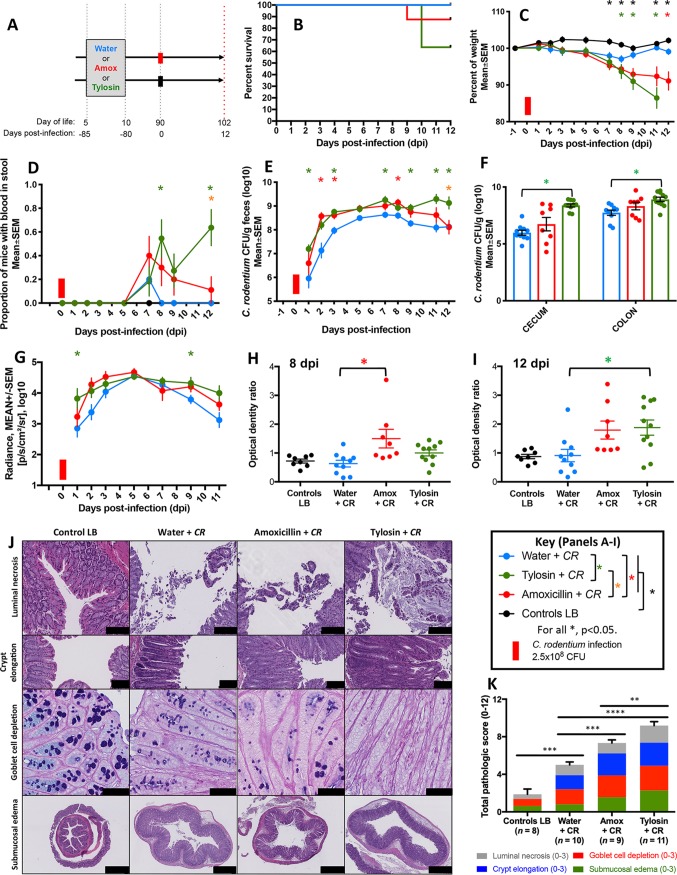
Characteristics of mice challenged with C. rodentium (or LB) 80 days after antibiotic or water exposure. (A) Study design. Five-day-old pups received a 5-day course of tylosin (*n* = 14), amoxicillin (*n* = 13), or water (*n* = 12). Eighty days later, pups were challenged with *luxCDABE* C. rodentium (water + *CR* [*n* = 10; blue], tylosin + *CR* [*n* = 11; green], and amoxicillin + *CR* [*n* = 10; red]) or with LB (water + LB [*n* = 2], tylosin + LB [*n* = 3], or amoxicillin + LB [*n* = 3]; black). (B) Percent surviving by exposure group. (C) Body weight over time, calculated as a percentage of prechallenge weight. For the tylosin group, analysis was censored at day 12 due to multiple deaths. (D) Proportion of mice with fecal occult blood. (E and F) Quantitation of C. rodentium, by culture, of feces (over time) and colon and cecum (at sacrifice). (G) Quantitation of C. rodentium by *in vivo* bioluminescent imaging. (H and I) Anti-C. rodentium IgM antibody levels 8 and 12 days postinfection (dpi), respectively. (J) Representative H&E-stained (or, in the third row, AB/PAS-stained) distal colonic sections (scale bars: for luminal necrosis, 100 μm; for crypt elongation, 100 μm; for goblet cell depletion, 50 μm; and for submucosal edema, 500 μm). (K) Cumulative histopathology scores (means ± SEM), calculated by evaluating luminal necrosis, crypt elongation, goblet cell depletion, and submucosal edema. **, *P* < 0.01; ***, *P* < 0.001; ****, *P* < 0.0001. For symbols, see key.

Since the consequences of early-life antibiotics may persist into adulthood (28, 30, 35), we next examined the severity of C. rodentium-induced colitis exactly as before, but now the interval to pathogen challenge was 80 days and mice were sacrificed 12 days postchallenge (Fig. 5A). Compared to that of the antibiotic-naive mice, those exposed to antibiotics trended toward decreased survival (*P* = 0.07 for the antibiotic groups combined, log rank test) (Fig. 5B) and showed greater weight loss during C. rodentium infection (Fig. 5C). The mice exposed to tylosin in early life more frequently had fecal occult blood (Fig. 5D) and had higher fecal C. rodentium loads (Fig. 5E), higher cecal and colonic C. rodentium counts (Fig. 5F), and higher *in vivo* C. rodentium levels (Fig. 5G and Fig. S2B). The antibiotic-exposed mice developed higher IgM responses to C. rodentium infection than the antibiotic-naive mice (Fig. 4H and I). Among the C. rodentium-infected mice, those exposed to antibiotics had more extensive colonic tissue injury (which was more severe in tylosin-exposed mice than amoxicillin-exposed mice) than did antibiotic-naive mice (Fig. 5J and K).

**FIG 6:**
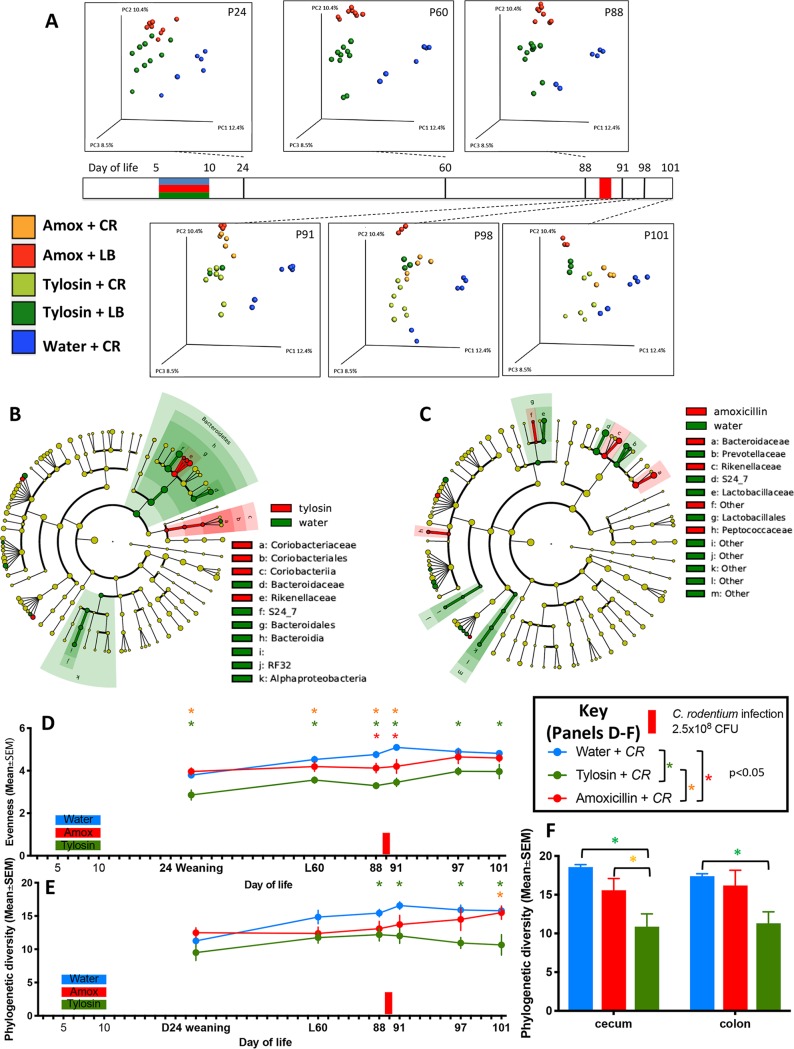
Gut microbiota characteristics of mice challenged with C. rodentium (or LB) 80 days after antibiotic or water exposure. (A) Study design (see the legend to Fig. 5A). Shown is an unweighted UniFrac analysis of fecal specimens visualized PCoA (for statistical significance, see Table S3). This cohort excluded a group treated with water plus LB. The three components explain 31.3% of the total variance. (B and C) LefSe analysis, with cladograms showing significantly differential taxa between indicated treatment groups on day 88 (2 days prior to C. rodentium challenge). Shading indicates significant overrepresentation of the indicated taxa (*P* < 0.05; LDA > 2). (D and E) Fecal pellets were collected at the times shown in bold, and α-diversity using the phylogenetic diversity (PD whole tree) metric (D) or Shannon index of evenness (E) is shown. (F) Cecal and colonic contents were collected at sacrifice, and α-diversity using the phylogenetic diversity (PD whole tree) metric is shown. Samples were obtained 12 days after C. rodentium challenge from mice preexposed to water (blue), amoxicillin (red), or tylosin (green). For symbols, see key.

Seventy-eight days following the antibiotic exposure and 2 days prior to C. rodentium or LB (control) inoculation, the exposed mice significantly differed from the antibiotic-naive mice in both microbial community structure (β-diversity) (Fig. 6A) and taxon composition (Fig. 6B and C), confirming that a single early-life antibiotic course had long-lasting effects on the host’s microbiota (28, 30). C. rodentium challenge further affected β-diversity (Fig. 6A). Both prior to and following C. rodentium challenge, early-life tylosin exposure significantly reduced gastrointestinal α-diversity compared to that of antibiotic-naive mice, while amoxicillin exposure had an intermediate effect (Fig. 6D to F). This experiment reveals that early-life antibiotic exposure has effects on both host microbiota and subsequent C. rodentium infection severity in adult mice.

10.1128/mBio.02820-19.6 TABLE S3 Summary of Adonis and ANOSIM testing of unweighted UniFrac distances, related to Fig. 6A. Download Table S3, DOCX file, 0.02 MB. Copyright © 2019 Roubaud-Baudron et al. 2019 Roubaud-Baudron et al. This content is distributed under the terms of the Creative Commons Attribution 4.0 International license.

### The antibiotic-perturbed microbiota transfers enhanced susceptibility to colitis after subsequent C. rodentium challenge.

**FIG 7:**
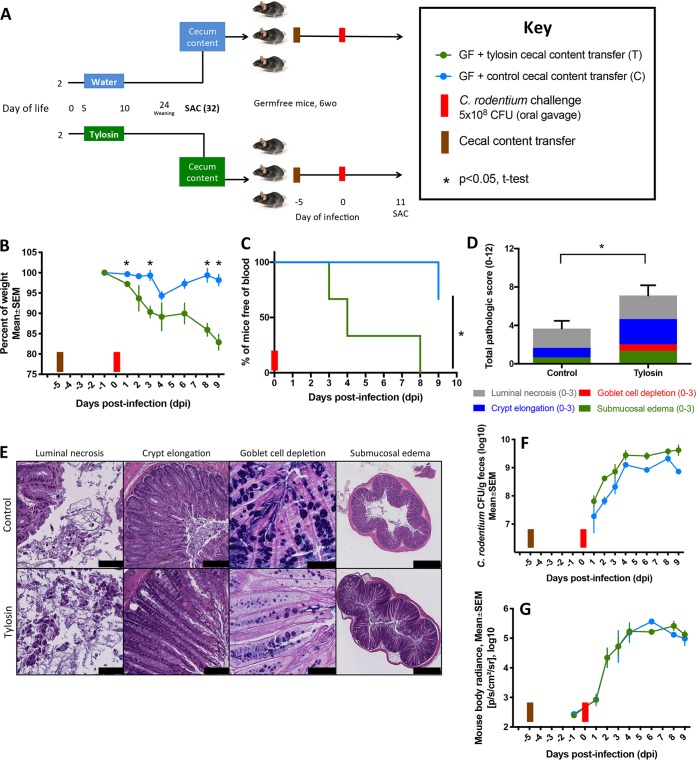
Transfer experiment: characteristics of recipient mice challenged with C. rodentium after gavage with tylosin-perturbed or control cecal contents. (A) Study design. Five-day-old conventionally raised mice received a 5-day course of tylosin (*n* = 2) or water (*n* = 2) and were sacrificed 22 days later. Cecal contents from these mice were transferred by gavage to 6-week-old germfree mice (*n* = 3/group). Five days later, these recipient mice were challenged by *luxCDABE* C. rodentium. (B) Body weight over time, calculated as a percentage of prechallenge weight. (C) Kaplan-Meier curve assessed the occurrence of fecal occult blood. (D) Cumulative histopathology scores (means ± SEM)—calculated by evaluating luminal necrosis, crypt elongation, goblet cell depletion, and submucosal edema—in the recipient mice gavaged with control or tylosin-perturbed cecal contents. (E) Representative H&E-stained (or, in the third column, AB/PAS-stained) distal colonic sections from recipient mice at sacrifice (scale bars: for luminal necrosis, 50 μm; for crypt elongation, 100 μm; for goblet cell depletion, 50 μm; and for submucosal edema, 500 μm). (F) Quantitation of C. rodentium in fecal culture. (G) Quantitation of C. rodentium by *in vivo* bioluminescent imaging. For symbols, see key.

In prior studies, the antibiotic-perturbed microbiota harvested 2 days after tylosin exposure ceased was sufficient to transfer immunological phenotypes to germfree mice (30). We now sought to examine whether the antibiotic-perturbed microbiota, in the current models, would lead to differential responses to C. rodentium challenge. To this end, we harvested the cecal contents of 32-day-old mice that had received tylosin or not (control) between days 5 and 10 of life. We then gavaged 6-week-old germfree mice with these tylosin-perturbed or control cecal contents and 5 days later challenged them with C. rodentium (Fig. 7A). Mice were sacrificed at 9 dpi since by this time, recipients of the tylosin-perturbed microbiota had experienced substantial weight loss compared to that of controls (Fig. 7B). Recipients of the tylosin-perturbed microbiota also suffered more severe colitis than did controls, with more frequent fecal occult blood (*P* = 0.026, log rank test) (Fig. 7C) and more extensive colonic tissue injury (Fig. 7D and E). Fecal C. rodentium levels, but not *in vivo* C. rodentium levels, trended higher in the recipients of the perturbed microbiota (Fig. 7F and G).

**FIG 8:**
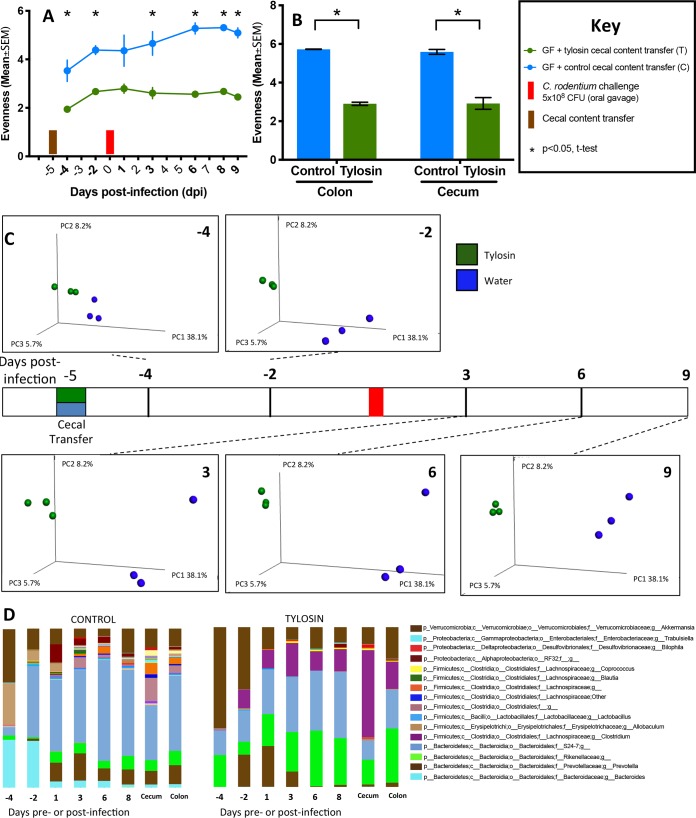
Transfer experiment: gut microbiota characteristics of recipient mice after gavage with tylosin-perturbed or control cecal contents and subsequent C. rodentium challenge. (A) Study design (see the legend to Fig. 7A). Fecal pellets were collected at the times shown in bold, and α-diversity using the Shannon index of evenness is shown. (B) α-Diversity of colonic and cecal contents at sacrifice using the Shannon index of evenness is shown. (C) Unweighted UniFrac analysis of fecal specimens visualized by PCoA. The three components explain 52% of the total variance. Statistical analysis of intergroup UniFrac distances was performed by Adonis test, with *P* values shown. (D) Relative abundances of taxa (present at >1% at the species level) in recipients of control or tylosin-perturbed cecal contents (control and tylosin, respectively). Days pre- or postinfection refer to fecal samples; cecum and colon were obtained at sacrifice. For symbols, see key.

Compared to that in the control microbiota recipients, α-diversity in fecal specimens obtained both prior to and following C. rodentium challenge was decreased in the recipients of the tylosin-perturbed microbiota (Fig. 8A) and in their colonic and cecal contents obtained at sacrifice (Fig. 8B). The fecal microbiota of the two groups differed significantly in β-diversity, and the cecal microbiota showed specific taxonomic differences, which persisted after the C. rodentium challenge (Fig. 8C and D). This experiment reveals that 23 days following cessation of the antibiotic exposure, the antibiotic-perturbed microbiota continues to be sufficient to enhance C. rodentium-induced colitis.

### Mice exposed to early-life antibiotics had decreases in *Bacteroidia* that persisted up to 78 days postexposure.

To determine whether specific bacterial species are associated with protection against C. rodentium, in two experiments we compared microbiota compositions between the antibiotic-exposed and control groups 21 days (Fig. 4D) and 78 days (Fig. 6B and C) after the exposure ended, in both cases immediately before the C. rodentium challenge. In both experiments, the antibiotic-exposed mice had reductions of *Bacteroidia* members (especially of the S24-7 family), and of *Alphaproteobacteria* and Deltaproteobacteria members (Fig. 4D and Fig. 6B and C); the effects of tylosin exposure were more pronounced than with amoxicillin, as reported previously (28). The S24-7 family was similarly depleted in the tylosin-perturbed inocula that had been used in the transfer studies (Fig. 8D). These results provide evidence associating *Bacteroidales* with protection against C. rodentium.

## DISCUSSION

Antibiotic use, particularly in young children, is occurring at high levels (31, 36, 37). Perturbations of the microbiota induced by antibiotic exposure can be long term (38–41) and also can compromise host mucosal immunity (24). Both clinical and epidemiologic studies provide evidence that antibiotic use, especially in young children, increases susceptibility to gastrointestinal infections in the postantibiotic period (32, 42).

In this study, using a model system, we examined the effects of early-life antibiotic exposure on the host microbiota and on the course of bacterial colitis induced after antibiotic cessation. Since early life is critical to microbiota development in humans (43–45), and microbiota perturbations during this critical window may be more long lasting (46), we studied whether the durability of these effects is enhanced in mice exposed to antibiotics as pups.

Our first model involved mice exposed to tylosin as young adults and challenged with C. rodentium immediately after the course was completed. The resulting microbiota perturbations and more severe colitis were consistent with studies involving exposure to a different antibiotic (metronidazole) prior to C. rodentium-induced colitis (14) and with studies in the context of other antibiotic classes and other gastrointestinal pathogens (13, 47–49).

Since early-life antibiotic exposure induces long-term effects on host microbiota (28, 30), and microbiota composition directly influences the outcome of C. rodentium infection (22, 23), we asked whether susceptibility to C. rodentium represents another durable consequence of early-life antibiotic exposure. The antibiotics used in the current models—tylosin (macrolide) and amoxicillin (β-lactam)—represent the two classes of antibiotics most frequently prescribed to children (37). Moreover, they were dosed at levels that are therapeutic in mice (50–52) and consistent with recommendations for the treatment of acute infections in children (53, 54).

In agreement with our prior studies (28, 30), the microbiota perturbations induced by early-life antibiotics remained present up to the longest interval studied (now up to 80 days postexposure). The severity and durability of these perturbations were greater for the macrolide than the β-lactam, as reported for both mice (28) and children (38). That the tylosin-exposed mice developed more severe disease than the amoxicillin-exposed hosts links the extent of microbiota perturbation with colitis severity, a finding consistent with studies of postantibiotic gastroenteritis severity correlating with the antibiotic dose (47).

Although differences in mouse vendors and strains have been causally associated with interhost differences in C. rodentium infection susceptibility (22, 23), the enhanced colitis we observed could have directly resulted from immunomodulatory effects of macrolides early in life (55, 56) rather than from microbiota-mediated changes. Our transfer experiment examined that issue. Compared to control recipients, the microbiota in recipients of the tylosin-perturbed inocula showed diminished community richness and altered community structure persisting throughout the posttransfer period, highlighting the durability of the early-life tylosin-induced microbiota alterations (30). That the recipients of the antibiotic-perturbed inocula experienced more severe infection upon C. rodentium challenge than the control recipients confirms the causal role of the perturbed microbiota in worsening the colitis.

Multiple mechanisms may explain how the antibiotic-perturbed microbiota increases host susceptibility to C. rodentium infection. First, antibiotic-induced reductions in the total microbiota burden may directly enable C. rodentium to colonize at higher levels (57). However, total 16S microbial densities in mice exposed to early-life antibiotics return to normal almost immediately (30), indicating the importance of microbiota composition rather than number, especially for the remote challenges. *Proteobacteria* members known to compete with C. rodentium were reduced by antibiotics, which may facilitate its mucosal colonization (58). Antibiotics alter production of microbiota metabolites sensed by C. rodentium, like indole, which aid the organism in discriminating lumen and epithelial surfaces (59). Ampicillin exposure also decreases short-chain fatty acid (SCFA)-producing bacteria permitting *Enterobacteriaceae* expansion (7), providing another mechanism for colonization resistance.

Second, through altering microbiota composition, antibiotics may reduce goblet cell mucus production; a thinner mucus layer allows increased C. rodentium attachment to the epithelium (14, 60). The greater severity of C. rodentium colitis in mice genetically deficient in mucin production than in wild-type hosts highlights the protective importance of the mucus layer (57). However, regardless of mechanism, the effects of antibiotic exposure on mucus production should be immediate and short term; thus, a direct antibiotic role is not compatible with a model in which C. rodentium challenge occurs months after the early-life antibiotic exposure has ended. However, an indirect role of antibiotics in host mucus production—in which the antibiotic-perturbed microbiota serves as the intermediary factor—is consistent with our observations. We have confirmed that a single early-life antibiotic course led to long-lasting reductions of *Bacteroidales*—especially of the S24-7 family (29, 30), which are known to stimulate colonic mucus production (61), perhaps by recruiting interleukin 6 (IL-6)-producing intraepithelial lymphocytes (62). S24-7 family abundance has been associated with a more resistant mucus layer (63).

Third, by depleting the native microbiota, antibiotics may reduce production of IL-22 and antimicrobial peptides, which are important innate immune defenses against C. rodentium (23); however, a direct antibiotic role implies that these levels return to normal after the exposure ceases.

Fourth, early-life tylosin exposure decreases colonic T_H_17 cells (30), as we reconfirmed, and in other experimental models, we found that the T_H_17 cell reduction persisted for >24 days following a similar antibiotic exposure (C. Ozkul and V. Ruiz, unpublished data). That mice deficient in segmented filamentous bacteria (“*Candidatus* Savagella”), a commensal species that induces intestinal T_H_17 cell differentiation, were more susceptible to C. rodentium-induced colitis underscores the importance of these cells in C. rodentium resistance (22).

Our study is limited, since although we have identified a causal role for early-life antibiotic-induced microbiota perturbation in the outcome of C. rodentium infection, we have not identified the exact mechanisms enabling C. rodentium to flourish. Our study also is limited to the effects of two antibiotics. While exposure to many antibiotic classes enhances C. rodentium-induced colitis, exposure to streptomycin does not (14), indicating that the selective effects on particular microbial taxa may be important. By suppressing susceptible organisms, streptomycin selects for *Bacteroidetes* and is protective in murine DSS (dextran sulfate sodium)-induced colitis, limiting *Enterobacteriaceae* blooms (64); although the ecological effects of streptomycin exposure are complex, that finding is consistent with a role of anaerobes in controlling C. rodentium populations.

Although we cannot exclude the possibility that early-life antibiotic exposure may reset immunological tone that directly leads to enhanced colitis susceptibility, the ability of the transferred microbiota to confer enhanced colitis indicates that the perturbed microbiota of early life is at least causally involved. Studies involving the transfer of immunocytes from antibiotic-exposed mice to immunodeficient mice will be required to test specific immunological hypotheses.

In conclusion, early-life antibiotic exposure in mice induces a long-lasting state of susceptibility to the consequences of C. rodentium infection. Extrapolating these results to humans suggests that antibiotic courses given to children may increase susceptibility to subsequent infections, even ones unrelated to those for which the antibiotic was prescribed (65). Restoring the critical species that were reduced or depleted by early-life antibiotics could alleviate such effects.

## MATERIALS AND METHODS

### Mice.

Four separate experiments were conducted. The first experiment involved 21-day-old female C57BL6/J mice obtained from the Jackson Laboratory (Bar Harbor, ME). These mice were exposed to tylosin or water from days 27 to 32 of life (Fig. 1A). The second and third experiments involved 6-week-old male and female C57BL/6J obtained from the Jackson Laboratory, which were bred to produce litters of newborn pups. Mothers were exposed to amoxicillin, tylosin, or neither in their drinking water (*n* = 6, *n* = 6, and *n* = 5, respectively), and their pups were then exposed via their mother’s milk (*n* = 26, *n* = 27, and *n* = 27, respectively). When pups reached 24 days of age, litters were weaned, and mice were separated by sex and by antibiotic treatment. In assembling the groups for challenge and follow-up, we maximized mixing of the pups between litters to minimize cage effects. Pups from these groups were then challenged with C. rodentium (*n* = 10/treatment group) or LB (as a control; *n* = 3/treatment group) either 23 days later (in the second experiment [Fig. 3A]) or 80 days later (in the third experiment [Fig. 5A]). The fourth experiment (Fig. 7A) involved wild-type C57BL/6 mice that were exposed to tylosin (*n* = 2) or water (*n* = 2) during days 5 to 10 of life (through their mother’s milk), sacrificed at day 32 of life, and used as cecal content donors. Female germfree C57BL/6 mice at the age of 6 weeks (*n* = 6) were used as recipients of either tylosin-perturbed (*n* = 3) or control (*n* = 3) cecal contents. All mice were maintained on a 12-h light/dark cycle and allowed *ad libitum* access to food and water in a level 2 animal facility. Mice were sacrificed via carbon dioxide asphyxiation and cervical dislocation.

### Ethics statement.

All animal experimentation was approved by the New York University School of Medicine Institutional Animal Care and Use Committee (IACUC protocol no. S15-01484) in accordance with the National Institutes of Health’s *Public Health Service Policy on Humane Care and Use of Laboratory Animals* (70) and the National Research Council of the National Academy of Sciences’ *Guide for the Care and Use of Laboratory Animals* (71).

### Antibiotic exposures.

Tylosin tartrate and amoxicillin trihydrate (Sigma-Aldrich, St. Louis, MO) were dissolved in distilled deionized water at concentrations of 0.333 and 0.167 mg/ml, respectively. Mice were exposed to antibiotics through their drinking water or their mother’s milk, as described previously (28, 30). The serum half-lives of tylosin and amoxicillin both are less than 1 h (28).

### Citrobacter rodentium infection.

We used a bioluminescent strain of C. rodentium that contained a chromosomal promoterless *luxCDABE* operon from the nematode symbiont Photorhabdus luminescens and a kanamycin resistance cassette (pUTmini-Tn*5* luxKm2) (72). A single C. rodentium colony was inoculated into Luria broth (LB) and grown overnight (at 37°C and 150 rpm). As confirmed by dilution plating, mice were challenged by oral gavage with 0.1 ml of LB containing between 2.5 × 10^8^ and 5 × 10^8^ CFU of C. rodentium.

To establish an enteric infection challenge model, we asked whether a single C. rodentium gavage was sufficient or whether two gavages was optimal. Since there were no significant differences between the two approaches in mouse weight, fecal occult blood, or C. rodentium quantitation (Fig. S1A to D), we used a single gavage in subsequent experiments. Mice also were monitored for morbidity and mortality following C. rodentium challenge. Mice were weighed every 2 days after challenge and were euthanized if signs of extreme distress (>20% loss of prechallenge body weight, hunched posture, inactivity, or seizure) were present. The presence of fecal occult blood was determined using Hemoccult (Beckman Coulter, Indianapolis, IN). Fecal, cecal, and colonic specimens were collected and either diluted in phosphate-buffered saline (PBS) for C. rodentium quantitation or frozen at –80°C for microbiota analyses. Every other day during the postinfection period, mice were anesthetized with 2% isoflurane mixed with 2% oxygen, imaged using IVIS (PerkinElmer, Santa Clara, CA), and then returned to their cages after imaging. Images were analyzed with Living Image v4.5.2 software (PerkinElmer) (72), and the average radiance for each mouse was obtained.

### Citrobacter rodentium quantitation.

Fecal pellets collected from individual mice were weighed, homogenized in 1 ml of PBS with autoclaved beads, serially diluted, inoculated (in duplicate) on MacConkey agar plates containing kanamycin (50 mg/ml), and incubated at 37°C. Bacterial colonies were enumerated after 24 h.

### Histopathological scoring.

Formalin-fixed, paraffin-embedded distal colonic tissue sections (5 μm) were obtained from mice upon sacrifice and were stained with hematoxylin and eosin (H&E) or with alcian blue (AB)/periodic acid-Schiff (PAS) (Poly Scientific R&D Corp., Bay Shore, NY) for goblet cell visualization. Stained slides were scanned for visualization on an SCN400F instrument (Leica Biosystems Inc, Buffalo Grove, IL). Tissue sections were scored, in a blinded fashion, using the following criteria adapted from similar studies (48, 60). Each criterion was scored on a scale of 0 to 3, for a maximum cumulative score of 12. The upper limits of the first, second, and third quartiles of the complied measurements for criteria ii to iv were calculated and used as the thresholds for the minimum mean measurement required for a scaled score of 1, 2, or 3, respectively. Scored characteristics were as follows. (i) Luminal necrosis, i.e., the presence or density of necrotic epithelial cells in the lumen, was scored as follows: 0, absent; 1, scant; 2, moderate; and 3, dense. (ii) Crypt elongation, i.e., the heights of 20 well-oriented, randomly selected crypts, was measured, and the mean crypt length was scored as follows: 0, ≤209 μm; 1, 210 to 258 μm; 2, 259 to 318 μm; and 3, ≥319 μm. (iii) For goblet cell depletion, 16 high-power fields (HPFs) (rectangular fields of dimensions 210 μm by 210 μm) were randomly selected, as described previously (48); within each HPF, goblet cell numbers and volumes were calculated using ImageJ v1.50 software (73). The goblet cell counts per HPF were then normalized to the mean goblet cell volume, and the mean goblet cell count per HPF was scored as follows: 0, ≥74; 1, 39 to 73; 2, 15 to 38; and 3, ≤14). (iv) For submucosal edema, the percent area of the intestinal wall occupied by the submucosa was calculated using ImageJ v1.50 software and scored as follows: 0, ≤3.2%; 1, 3.3 to 5.7%; 2, 5.8 to 9.3%; and 3, ≥9.4%.

### Microbiota transfer.

Cecal contents were collected from donor mice as described above and the contents divided, with half immediately immersed in prereduced anaerobic dental transport medium (Anaerobe Systems, Morgan Hill, CA) and then frozen at –80°C, as described previously (66). Upon thawing under anaerobic conditions, the cecal contents from 2 mice/group (tylosin preexposed or control) were pooled and further diluted in dental transport medium. Germfree mice received this suspension (150 μl) via oral gavage and 5 days later were challenged by C. rodentium, as described above.

### Determination of anti-Citrobacter rodentium antibody titers in serum.

Peripheral blood was collected from mice before C. rodentium challenge, and at 8 and 11 dpi, by submandibular puncture. Blood samples were centrifuged at 2,000 × *g* for 10 min and sera stored at –80°C. Specific antibodies to C. rodentium antigens were quantitiated by enzyme-linked immunosorbent assay (ELISA) using a sonicated overnight culture of C. rodentium as the antigen source, and protein concentrations were measured using a bicinchoninic acid (BCA) protein assay kit (Fisher Scientific). All assays were performed in duplicate with positive and negative internal controls. The antigen was diluted in 0.5 M carbonate buffer (pH 9.6) for a protein concentration of 10 μg/ml. Aliquots (0.1 ml) were added to wells of flat-bottom Immulon 2 plates (Dynatech Laboratories, Alexandria, VA) and incubated overnight at room temperature. Nonspecific binding was prevented by incubating plates with PBS buffer with 0.1% gelatin for 4 h at 37°C. After incubation, plates were washed 3 times and 0.1-ml volumes of serum samples diluted to 1:100 in PBS were added. After incubation at 37°C for 1 h, plates were washed 3 times and 0.1-ml volumes of peroxidase conjugates of goat anti-human IgG (Thermo Fisher, Waltham, MA) or IgM (Invitrogen) diluted to 1:1,000 were added. Horseradish peroxidase (HRP) substrate was added and the colorimetric reaction was quantified by MRX Revelation (Dynex Technologies, Chantilly, VA).

### DNA extraction, library preparation, and microbial community analysis.

DNA was extracted from fecal, cecal, and colonic specimens using a MoBio 96-well extraction kit. For amplicon library generation, the 16S rRNA V4 region was amplified with gene-specific primers, as described previously (66). The reverse amplification primers contained a 12-bp Golay barcode for multiplexed sequencing runs that support pooling >1,000 samples. Amplicons were prepared in triplicate, pooled, and quantified. The 254-bp V4 region was sequenced using the Ilumina MiSeq 2 × 150-bp platform. Operational taxonomic units (OTUs) were picked using an open reference picking strategy and Greengenes 13_8 for reference. Downstream processing of 16S rRNA sequences was performed using the QIIME 1.9.1 pipeline (67). Sequences were quality filtered with a Phred score of 20, chimeras were removed (ChimeraSlayer), and samples with a sequence depth of <600 were excluded. Filtered reads were clustered into 97% identity OTUs using the program UCLUST, followed by taxonomy assignment using the RDP Classifier. The phylogenetic tree and abundance tables generated were used to calculate unweighted and weighted UniFrac diversity indices. The OTU absolute-abundance tables were extracted from the pipeline for further analysis using the phyloseq package in the R statistical programming environment (68). Taxon compositions were compared using LefSe (69).

### Isolation of colonic lamina propria lymphocytes.

The large intestine was excised, placed in digestion medium containing 1 mM dithiothreitol (DTT) and 1 mM EDTA in calcium- and magnesium-free Hanks balanced salt solution (HBSS) supplemented with 2% fetal calf serum (FCS), and subsequently treated with collagenase IV-DNase digestion mix (0.5 mg/ml of collagenase IV and 200 μg/ml of DNase). Lymphocytes were enriched using a 40%/80% discontinuous Percoll (GE Lifesciences, Pittsburgh, PA) gradient. Lymphocytes derived from mesenteric lymph nodes were isolated through mechanical processing using a sterile 70-μm cell strainer (Corning, Corning, NY). Cells were stimulated with phorbol 12-myristate 13-acetate (PMA) and ionomycin for 4 h at 37°C in the presence of brefeldin A (GolgiPlug; BD Biosciences, San Jose, CA). Following stimulation, cells were stained with LIVE/DEAD fixable aqua (Thermo Fisher) and antibody-fluorophore combinations (i.e., allophycocyanin [APC]-Cy7–CD45, T cell receptor β [TCR-β]-fluorescein isothiocyanate [FITC], and CD4-V500 [BD Biosciences] and CD8-BV650, Foxp3–phycoerythrin [PE]-Cy7, IL-17–PE, and gamma interferon [IFN-γ]–FITC [eBioscience, San Diego, CA]) and then fixed with fix/perm (eBioscience) according to the manufacturer’s instructions. Cells were acquired on an LSRII flow cytometer (BD Biosciences) and analyzed with FlowJo software (Tree Star, Ashland, OR); >100,000 events were collected for each sample. Samples with yields of <10,000 viable events were excluded from analysis.

### Statistical analysis.

Data are expressed as means ± standard errors of the means (SEM). Unless otherwise indicated, group means were compared by Kruskal-Wallis test with a *post hoc* test (Dunn’s) for multiple comparisons, using Prism 8 software (GraphPad, La Jolla, CA).

## References

[1] Schaedler RW, Dubos R, Costello R 1965 The development of the bacterial flora in the gastrointestinal tract of mice. J Exp Med 122:59–66. doi:10.1084/jem.122.1.59. 14325473 PMC2138024

[2] Savage DC, Dubos R, Schaedler RW 1968 The gastrointestinal epithelium and its autochthonous bacterial flora. J Exp Med 127:67–76. doi:10.1084/jem.127.1.67. 4169441 PMC2138434

[3] Gill SR, Pop M, Deboy RT, Eckburg PB, Turnbaugh PJ, Samuel BS, Gordon JI, Relman DA, Fraser-Liggett CM, Nelson KE 2006 Metagenomic analysis of the human distal gut microbiome. Science 312:1355–1359. doi:10.1126/science.1124234. 16741115 PMC3027896

[4] Ubeda C, Djukovic A, Isaac S 2017 Roles of the intestinal microbiota in pathogen protection. Clin Transl Immunology 6:e128. doi:10.1038/cti.2017.2. 28243438 PMC5311919

[5] Abt MC, Pamer EG 2014 Commensal bacteria mediated defenses against pathogens. Curr Opin Immunol 29:16–22. doi:10.1016/j.coi.2014.03.003. 24727150 PMC4132187

[6] Brown RL, Clarke TB 2017 The regulation of host defences to infection by the microbiota. Immunology 150:1–6. doi:10.1111/imm.12634. 27311879 PMC5221693

[7] Sorbara MT, Dubin K, Littmann ER, Moody TU, Fontana E, Seok R, Leiner IM, Taur Y, Peled JU, van den Brink MRM, Litvak Y, Baumler AJ, Chaubard JL, Pickard AJ, Cross JR, Pamer EG 2019 Inhibiting antibiotic-resistant Enterobacteriaceae by microbiota-mediated intracellular acidification. J Exp Med 216:84–98. doi:10.1084/jem.20181639. 30563917 PMC6314524

[8] Savage DC, Dubos R 1968 Alterations in the mouse cecum and its flora produced by antibacterial drugs. J Exp Med 128:97–110. doi:10.1084/jem.128.1.97. 5662019 PMC2138511

[9] Modi SR, Collins JJ, Relman DA 2014 Antibiotics and the gut microbiota. J Clin Invest 124:4212–4218. doi:10.1172/JCI72333. 25271726 PMC4191029

[10] Bohnhoff M, Drake BL, Miller CP 1954 Effect of streptomycin on susceptibility of intestinal tract to experimental Salmonella infection. Proc Soc Exp Biol Med 86:132–137. doi:10.3181/00379727-86-21030. 13177610

[11] Miller CP, Bohnhoff M, Rifkind D 1956 The effect of an antibiotic on the susceptibility of the mouse’s intestinal tract to Salmonella infection. Trans Am Clin Climatol Assoc 68:51–55; discussion, 55–58. 13486607 PMC2248925

[12] Debast SB, Bauer MP, Kuijper EJ 2014 European Society of Clinical Microbiology and Infectious Diseases: update of the treatment guidance document for Clostridium difficile infection. Clin Microbiol Infect 20(Suppl 2):1–26. doi:10.1111/1469-0691.12418. 24118601

[13] Becattini S, Littmann ER, Carter RA, Kim SG, Morjaria SM, Ling L, Gyaltshen Y, Fontana E, Taur Y, Leiner IM, Pamer EG 2017 Commensal microbes provide first line defense against Listeria monocytogenes infection. J Exp Med 214:1973–1989. doi:10.1084/jem.20170495. 28588016 PMC5502438

[14] Wlodarska M, Willing B, Keeney KM, Menendez A, Bergstrom KS, Gill N, Russell SL, Vallance BA, Finlay BB 2011 Antibiotic treatment alters the colonic mucus layer and predisposes the host to exacerbated Citrobacter rodentium-induced colitis. Infect Immun 79:1536–1545. doi:10.1128/IAI.01104-10. 21321077 PMC3067531

[15] Schauer DB, Falkow S 1993 Attaching and effacing locus of a Citrobacter freundii biotype that causes transmissible murine colonic hyperplasia. Infect Immun 61:2486–2492. 8500884 10.1128/iai.61.6.2486-2492.1993 PMC280873

[16] Collins JW, Keeney KM, Crepin VF, Rathinam VA, Fitzgerald KA, Finlay BB, Frankel G 2014 Citrobacter rodentium: infection, inflammation and the microbiota. Nat Rev Microbiol 12:612–623. doi:10.1038/nrmicro3315. 25088150

[17] Eckmann L 2006 Animal models of inflammatory bowel disease: lessons from enteric infections. Ann N Y Acad Sci 1072:28–38. doi:10.1196/annals.1326.008. 17057188

[18] Mundy R, MacDonald TT, Dougan G, Frankel G, Wiles S 2005 Citrobacter rodentium of mice and man. Cell Microbiol 7:1697–1706. doi:10.1111/j.1462-5822.2005.00625.x. 16309456

[19] Maaser C, Housley MP, Iimura M, Smith JR, Vallance BA, Finlay BB, Schreiber JR, Varki NM, Kagnoff MF, Eckmann L 2004 Clearance of Citrobacter rodentium requires B cells but not secretory immunoglobulin A (IgA) or IgM antibodies. Infect Immun 72:3315–3324. doi:10.1128/IAI.72.6.3315-3324.2004. 15155635 PMC415672

[20] Vallance BA, Deng W, Jacobson K, Finlay BB 2003 Host susceptibility to the attaching and effacing bacterial pathogen Citrobacter rodentium. Infect Immun 71:3443–3453. doi:10.1128/iai.71.6.3443-3453.2003. 12761129 PMC155702

[21] Ghosh S, Dai C, Brown K, Rajendiran E, Makarenko S, Baker J, Ma C, Halder S, Montero M, Ionescu VA, Klegeris A, Vallance BA, Gibson DL 2011 Colonic microbiota alters host susceptibility to infectious colitis by modulating inflammation, redox status, and ion transporter gene expression. Am J Physiol Gastrointest Liver Physiol 301:G39–G49. doi:10.1152/ajpgi.00509.2010. 21454446

[22] Ivanov II, Atarashi K, Manel N, Brodie EL, Shima T, Karaoz U, Wei D, Goldfarb KC, Santee CA, Lynch SV, Tanoue T, Imaoka A, Itoh K, Takeda K, Umesaki Y, Honda K, Littman DR 2009 Induction of intestinal Th17 cells by segmented filamentous bacteria. Cell 139:485–498. doi:10.1016/j.cell.2009.09.033. 19836068 PMC2796826

[23] Willing BP, Vacharaksa A, Croxen M, Thanachayanont T, Finlay BB 2011 Altering host resistance to infections through microbial transplantation. PLoS One 6:e26988. doi:10.1371/journal.pone.0026988. 22046427 PMC3203939

[24] Willing BP, Russell SL, Finlay BB 2011 Shifting the balance: antibiotic effects on host-microbiota mutualism. Nat Rev Microbiol 9:233–243. doi:10.1038/nrmicro2536. 21358670

[25] Mullineaux-Sanders C, Collins JW, Ruano-Gallego D, Levy M, Pevsner-Fischer M, Glegola-Madejska IT, Sågfors AM, Wong JLC, Elinav E, Crepin VF, Frankel G 2017 Citrobacter rodentium relies on commensals for colonization of the colonic mucosa. Cell Rep 21:3381–3389. doi:10.1016/j.celrep.2017.11.086. 29262319 PMC5746604

[26] Martinez I, Maldonado-Gomez MX, Gomes-Neto JC, Kittana H, Ding H, Schmaltz R, Joglekar P, Cardona RJ, Marsteller NL, Kembel SW, Benson AK, Peterson DA, Ramer-Tait AE, Walter J 2018 Experimental evaluation of the importance of colonization history in early-life gut microbiota assembly. Elife 7:e36521. doi:10.7554/eLife.36521. 30226190 PMC6143339

[27] Stewart CJ, Ajami NJ, O’Brien JL, Hutchinson DS, Smith DP, Wong MC, Ross MC, Lloyd RE, Doddapaneni H, Metcalf GA, Muzny D, Gibbs RA, Vatanen T, Huttenhower C, Xavier RJ, Rewers M, Hagopian W, Toppari J, Ziegler A-G, She J-X, Akolkar B, Lernmark A, Hyoty H, Vehik K, Krischer JP, Petrosino JF 2018 Temporal development of the gut microbiome in early childhood from the TEDDY study. Nature 562:583–588. doi:10.1038/s41586-018-0617-x. 30356187 PMC6415775

[28] Nobel YR, Cox LM, Kirigin FF, Bokulich NA, Yamanishi S, Teitler I, Chung J, Sohn J, Barber CM, Goldfarb DS, Raju K, Abubucker S, Zhou Y, Ruiz VE, Li H, Mitreva M, Alekseyenko AV, Weinstock GM, Sodergren E, Blaser MJ 2015 Metabolic and metagenomic outcomes from early-life pulsed antibiotic treatment. Nat Commun 6:7486. doi:10.1038/ncomms8486. 26123276 PMC4491183

[29] Zhang XS, Li J, Krautkramer KA, Badri M, Battaglia T, Borbet TC, Koh H, Ng S, Sibley RA, Li Y, Pathmasiri W, Jindal S, Shields-Cutler RR, Hillmann B, Al-Ghalith GA, Ruiz VE, Livanos A, van ’T Wout AB, Nagalingam N, Rogers AB, Sumner SJ, Knights D, Denu JM, Li H, Ruggles KV, Bonneau R, Williamson RA, Rauch M, Blaser MJ 2018 Antibiotic-induced acceleration of type 1 diabetes alters maturation of innate intestinal immunity. Elife 7:e37816. doi:10.7554/eLife.37816. 30039798 PMC6085123

[30] Ruiz VE, Battaglia T, Kurtz ZD, Bijnens L, Ou A, Engstrand I, Zheng X, Iizumi T, Mullins BJ, Muller CL, Cadwell K, Bonneau R, Perez-Perez GI, Blaser MJ 2017 A single early-in-life macrolide course has lasting effects on murine microbial network topology and immunity. Nat Commun 8:518. doi:10.1038/s41467-017-00531-6. 28894149 PMC5593929

[31] Rogawski ET, Platts-Mills JA, Seidman JC, John S, Mahfuz M, Ulak M, Shrestha SK, Soofi SB, Yori PP, Mduma E, Svensen E, Ahmed T, Lima AA, Bhutta ZA, Kosek MN, Lang DR, Gottlieb M, Zaidi AK, Kang G, Bessong PO, Houpt ER, Guerrant RL 2017 Use of antibiotics in children younger than two years in eight countries: a prospective cohort study. Bull World Health Organ 95:49–61. doi:10.2471/BLT.16.176123. 28053364 PMC5180352

[32] Rogawski ET, Westreich D, Becker-Dreps S, Adair LS, Sandler RS, Sarkar R, Kattula D, Ward HD, Meshnick SR, Kang G 2015 The effect of early life antibiotic exposures on diarrheal rates among young children in Vellore, India. Pediatr Infect Dis J 34:583–588. doi:10.1097/INF.0000000000000679. 25742244 PMC4431927

[33] Man WH, Clerc M, de Steenhuijsen Piters WAA, van Houten MA, Chu M, Kool J, Keijser BJF, Sanders EAM, Bogaert D 2019 Loss of microbial topography between oral and nasopharyngeal microbiota and development of respiratory infections early in life. Am J Respir Crit Care Med 200:760. doi:10.1164/rccm.201810-1993OC. 30883192

[34] Hoffmann C, Hill DA, Minkah N, Kirn T, Troy A, Artis D, Bushman F 2009 Community-wide response of the gut microbiota to enteropathogenic Citrobacter rodentium infection revealed by deep sequencing. Infect Immun 77:4668–4678. doi:10.1128/IAI.00493-09. 19635824 PMC2747949

[35] Jin S, Zhao D, Cai C, Song D, Shen J, Xu A, Qiao Y, Ran Z, Zheng Q 2017 Low-dose penicillin exposure in early life decreases Th17 and the susceptibility to DSS colitis in mice through gut microbiota modification. Sci Rep 7:43662. doi:10.1038/srep43662. 28272549 PMC5341569

[36] Hicks LA, Bartoces MG, Roberts RM, Suda KJ, Hunkler RJ, Taylor TH Jr, Schrag SJ 2015 US outpatient antibiotic prescribing variation according to geography, patient population, and provider specialty in 2011. Clin Infect Dis 60:1308–1316. 25747410 10.1093/cid/civ076

[37] Hersh AL, Shapiro DJ, Pavia AT, Shah SS 2011 Antibiotic prescribing in ambulatory pediatrics in the United States. Pediatrics 128:1053–1061. doi:10.1542/peds.2011-1337. 22065263

[38] Korpela K, Salonen A, Virta LJ, Kekkonen RA, Forslund K, Bork P, de Vos WM 2016 Intestinal microbiome is related to lifetime antibiotic use in Finnish pre-school children. Nat Commun 7:10410. doi:10.1038/ncomms10410. 26811868 PMC4737757

[39] Jernberg C, Lofmark S, Edlund C, Jansson JK 2007 Long-term ecological impacts of antibiotic administration on the human intestinal microbiota. ISME J 1:56–66. doi:10.1038/ismej.2007.3. 18043614

[40] Jakobsson HE, Jernberg C, Andersson AF, Sjolund-Karlsson M, Jansson JK, Engstrand L 2010 Short-term antibiotic treatment has differing long-term impacts on the human throat and gut microbiome. PLoS One 5:e9836. doi:10.1371/journal.pone.0009836. 20352091 PMC2844414

[41] Mangin I, Leveque C, Magne F, Suau A, Pochart P 2012 Long-term changes in human colonic Bifidobacterium populations induced by a 5-day oral amoxicillin-clavulanic acid treatment. PLoS One 7:e50257. doi:10.1371/journal.pone.0050257. 23209691 PMC3507739

[42] Ryan CA, Nickels MK, Hargrett-Bean NT, Potter ME, Endo T, Mayer L, Langkop CW, Gibson C, McDonald RC, Kenney RT, Puhr ND, McDonnell PJ, Martin RJ, Cohen ML, Blake PA 1987 Massive outbreak of antimicrobial-resistant salmonellosis traced to pasteurized milk. JAMA 258:3269–3274. doi:10.1001/jama.1987.03400220069039. 3316720

[43] Yatsunenko T, Rey FE, Manary MJ, Trehan I, Dominguez-Bello MG, Contreras M, Magris M, Hidalgo G, Baldassano RN, Anokhin AP, Heath AC, Warner B, Reeder J, Kuczynski J, Caporaso JG, Lozupone CA, Lauber C, Clemente JC, Knights D, Knight R, Gordon JI 2012 Human gut microbiome viewed across age and geography. Nature 486:222–227. doi:10.1038/nature11053. 22699611 PMC3376388

[44] Bäckhed F, Roswall J, Peng Y, Feng Q, Jia H, Kovatcheva-Datchary P, Li Y, Xia Y, Xie H, Zhong H, Khan MT, Zhang J, Li J, Xiao L, Al-Aama J, Zhang D, Lee YS, Kotowska D, Colding C, Tremaroli V, Yin Y, Bergman S, Xu X, Madsen L, Kristiansen K, Dahlgren J, Wang J, Jun W 2015 Dynamics and stabilization of the human gut microbiome during the first year of life. Cell Host Microbe 17:690–703. doi:10.1016/j.chom.2015.04.004. 25974306

[45] Yassour M, Vatanen T, Siljander H, Hamalainen AM, Harkonen T, Ryhanen SJ, Franzosa EA, Vlamakis H, Huttenhower C, Gevers D, Lander ES, Knip M, Group DS, Xavier RJ 2016 Natural history of the infant gut microbiome and impact of antibiotic treatment on bacterial strain diversity and stability. Sci Transl Med 8:343ra81. doi:10.1126/scitranslmed.aad0917. PMC5032909 27306663

[46] Bokulich NA, Chung J, Battaglia T, Henderson N, Jay M, Li H, A DL, Wu F, Perez-Perez GI, Chen Y, Schweizer W, Zheng X, Contreras M, Dominguez-Bello MG, Blaser MJ 2016 Antibiotics, birth mode, and diet shape microbiome maturation during early life. Sci Transl Med 8:343ra82. doi:10.1126/scitranslmed.aad7121. PMC5308924 27306664

[47] Sekirov I, Tam NM, Jogova M, Robertson ML, Li Y, Lupp C, Finlay BB 2008 Antibiotic-induced perturbations of the intestinal microbiota alter host susceptibility to enteric infection. Infect Immun 76:4726–4736. doi:10.1128/IAI.00319-08. 18678663 PMC2546810

[48] Barthel M, Hapfelmeier S, Quintanilla-Martínez L, Kremer M, Rohde M, Hogardt M, Pfeffer K, Rüssmann H, Hardt W-D 2003 Pretreatment of mice with streptomycin provides a Salmonella enterica serovar Typhimurium colitis model that allows analysis of both pathogen and host. Infect Immun 71:2839–2858. doi:10.1128/iai.71.5.2839-2858.2003. 12704158 PMC153285

[49] Buffie CG, Jarchum I, Equinda M, Lipuma L, Gobourne A, Viale A, Ubeda C, Xavier J, Pamer EG 2012 Profound alterations of intestinal microbiota following a single dose of clindamycin results in sustained susceptibility to Clostridium difficile-induced colitis. Infect Immun 80:62–73. doi:10.1128/IAI.05496-11. 22006564 PMC3255689

[50] Andes D, Craig WA 1998 In vivo activities of amoxicillin and amoxicillin-clavulanate against Streptococcus pneumoniae: application to breakpoint determinations. Antimicrob Agents Chemother 42:2375–2379. doi:10.1128/AAC.42.9.2375. 9736566 PMC105836

[51] Du X, Li C, Sun HK, Nightingale CH, Nicolau DP 2005 A sensitive assay of amoxicillin in mouse serum and broncho-alveolar lavage fluid by liquid-liquid extraction and reversed-phase HPLC. J Pharm Biomed Anal 39:648–652. doi:10.1016/j.jpba.2005.04.021. 15935600

[52] Lewicki J 2006 Tylosin: a review of pharmacokinetics, residues in food animals and analytical methods. United Nations Food and Agriculture Organization, Rome, Italy.

[53] Fonseca W, Hoppu K, Rey LC, Amaral J, Qazi S 2003 Comparing pharmacokinetics of amoxicillin given twice or three times per day to children older than 3 months with pneumonia. Antimicrob Agents Chemother 47:997–1001. doi:10.1128/aac.47.3.997-1001.2003. 12604533 PMC149282

[54] Nahata MC, Koranyi KI, Luke DR, Foulds G 1995 Pharmacokinetics of azithromycin in pediatric patients with acute otitis media. Antimicrob Agents Chemother 39:1875–1877. doi:10.1128/aac.39.8.1875. 7486938 PMC162845

[55] Iwanaga N, Nakamura S, Oshima K, Kajihara T, Takazono T, Miyazaki T, Izumikawa K, Yanagihara K, Sugawara A, Sunazuka T, Omura S, Kohno S 2015 Macrolides promote CCL2-mediated macrophage recruitment and clearance of nasopharyngeal pneumococcal colonization in mice. J Infect Dis 212:1150–1159. doi:10.1093/infdis/jiv157. 25767216

[56] Ratzinger F, Haslacher H, Poeppl W, Hoermann G, Kovarik JJ, Jutz S, Steinberger P, Burgmann H, Pickl WF, Schmetterer KG 2014 Azithromycin suppresses CD4(+) T-cell activation by direct modulation of mTOR activity. Sci Rep 4:7438. doi:10.1038/srep07438. 25500904 PMC4262884

[57] Bergstrom KS, Kissoon-Singh V, Gibson DL, Ma C, Montero M, Sham HP, Ryz N, Huang T, Velcich A, Finlay BB, Chadee K, Vallance BA 2010 Muc2 protects against lethal infectious colitis by disassociating pathogenic and commensal bacteria from the colonic mucosa. PLoS Pathog 6:e1000902. doi:10.1371/journal.ppat.1000902. 20485566 PMC2869315

[58] Kamada N, Kim YG, Sham HP, Vallance BA, Puente JL, Martens EC, Nunez G 2012 Regulated virulence controls the ability of a pathogen to compete with the gut microbiota. Science 336:1325–1329. doi:10.1126/science.1222195. 22582016 PMC3439148

[59] Kumar A, Sperandio V 2019 Indole signaling at the host-microbiota-pathogen interface. mBio 10:e01031-19. doi:10.1128/mBio.01031-19. 31164470 PMC6550529

[60] Wlodarska M, Thaiss CA, Nowarski R, Henao-Mejia J, Zhang JP, Brown EM, Frankel G, Levy M, Katz MN, Philbrick WM, Elinav E, Finlay BB, Flavell RA 2014 NLRP6 inflammasome orchestrates the colonic host-microbial interface by regulating goblet cell mucus secretion. Cell 156:1045–1059. doi:10.1016/j.cell.2014.01.026. 24581500 PMC4017640

[61] Burger-van Paassen N, Vincent A, Puiman PJ, van der Sluis M, Bouma J, Boehm G, van Goudoever JB, van Seuningen I, Renes IB 2009 The regulation of intestinal mucin MUC2 expression by short-chain fatty acids: implications for epithelial protection. Biochem J 420:211–219. doi:10.1042/BJ20082222. 19228118

[62] Kuhn KA, Schulz HM, Regner EH, Severs EL, Hendrickson JD, Mehta G, Whitney AK, Ir D, Ohri N, Robertson CE, Frank DN, Campbell EL, Colgan SP 2018 Bacteroidales recruit IL-6-producing intraepithelial lymphocytes in the colon to promote barrier integrity. Mucosal Immunol 11:357–368. doi:10.1038/mi.2017.55. 28812548 PMC5815964

[63] Jakobsson HE, Rodriguez-Pineiro AM, Schutte A, Ermund A, Boysen P, Bemark M, Sommer F, Backhed F, Hansson GC, Johansson ME 2015 The composition of the gut microbiota shapes the colon mucus barrier. EMBO Rep 16:164–177. doi:10.15252/embr.201439263. 25525071 PMC4328744

[64] Huang YL, Chassard C, Hausmann M, von Itzstein M, Hennet T 2015 Sialic acid catabolism drives intestinal inflammation and microbial dysbiosis in mice. Nat Commun 6:8141. doi:10.1038/ncomms9141. 26303108 PMC4560832

[65] Unger SA, Bogaert D 2017 The respiratory microbiome and respiratory infections. J Infect 74(Suppl 1):S84–S88. doi:10.1016/S0163-4453(17)30196-2. 28646967

[66] Cox LM, Yamanishi S, Sohn J, Alekseyenko AV, Leung JM, Cho I, Kim SG, Li H, Gao Z, Mahana D, Zarate Rodriguez JG, Rogers AB, Robine N, Loke P, Blaser MJ 2014 Altering the intestinal microbiota during a critical developmental window has lasting metabolic consequences. Cell 158:705–721. doi:10.1016/j.cell.2014.05.052. 25126780 PMC4134513

[67] Caporaso JG, Kuczynski J, Stombaugh J, Bittinger K, Bushman FD, Costello EK, Fierer N, Pena AG, Goodrich JK, Gordon JI, Huttley GA, Kelley ST, Knights D, Koenig JE, Ley RE, Lozupone CA, McDonald D, Muegge BD, Pirrung M, Reeder J, Sevinsky JR, Turnbaugh PJ, Walters WA, Widmann J, Yatsunenko T, Zaneveld J, Knight R 2010 QIIME allows analysis of high-throughput community sequencing data. Nat Methods 7:335–336. doi:10.1038/nmeth.f.303. 20383131 PMC3156573

[68] R Development Core Team. 2010 R Foundation for environment statistical computing. R Foundation for Statistical Computing, Vienna, Austria Retrieved from https://www.r-project.org/.

[69] Segata N, Izard J, Waldron L, Gevers D, Miropolsky L, Garrett WS, Huttenhower C 2011 Metagenomic biomarker discovery and explanation. Genome Biol 12:R60. doi:10.1186/gb-2011-12-6-r60. 21702898 PMC3218848

[70] National Institutes of Health. 2015 Public Health Service policy on humane care and use of laboratory animals. Office of Laboratory Animal Welfare, National Institutes of Health, Bethesda, MD.

[71] National Research Council. 2011 Guide for the care and use of laboratory animals, 8th ed National Academies Press, Washington, DC.

[72] Gibson DL, Ma C, Bergstrom KS, Huang JT, Man C, Vallance BA 2008 MyD88 signalling plays a critical role in host defence by controlling pathogen burden and promoting epithelial cell homeostasis during Citrobacter rodentium-induced colitis. Cell Microbiol 10:618–631. doi:10.1111/j.1462-5822.2007.01071.x. 17979981

[73] Schneider CA, Rasband WS, Eliceiri KW 2012 NIH Image to ImageJ: 25 years of image analysis. Nat Methods 9:671–675. doi:10.1038/nmeth.2089. 22930834 PMC5554542

