# Supplementary figures and images for: Long-Term Effects of Early-Life Antibiotic Exposure on Resistance to Subsequent Bacterial Infection
Source: mBio. 2019 Dec 24;10(6):e02820-19. doi: 10.1128/mBio.02820-19 (PMC6935859; doi:10.1128/mBio.02820-19)

Fig. S1

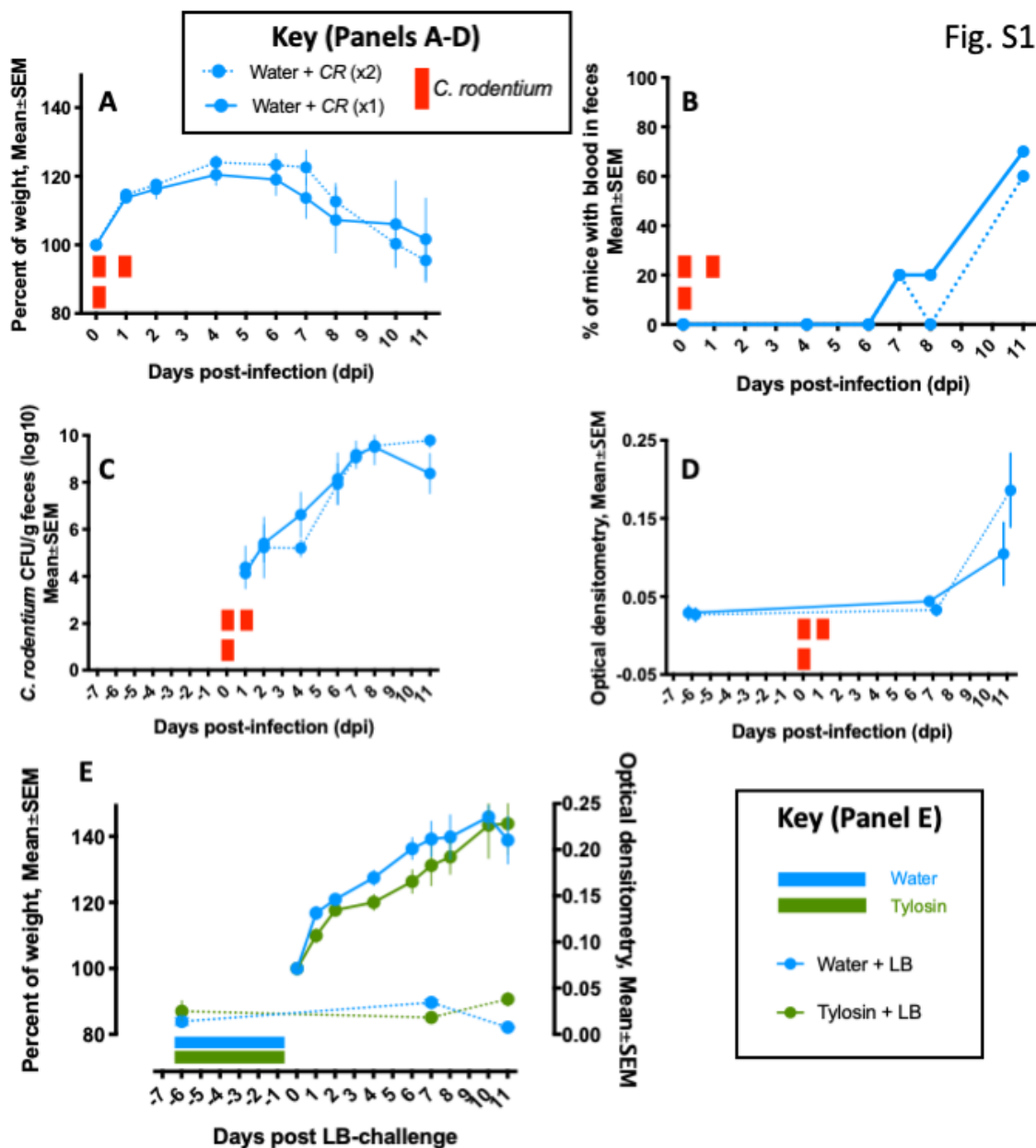

Supplement: FIG S1 [file mBio.02820-19-sf001.pdf]

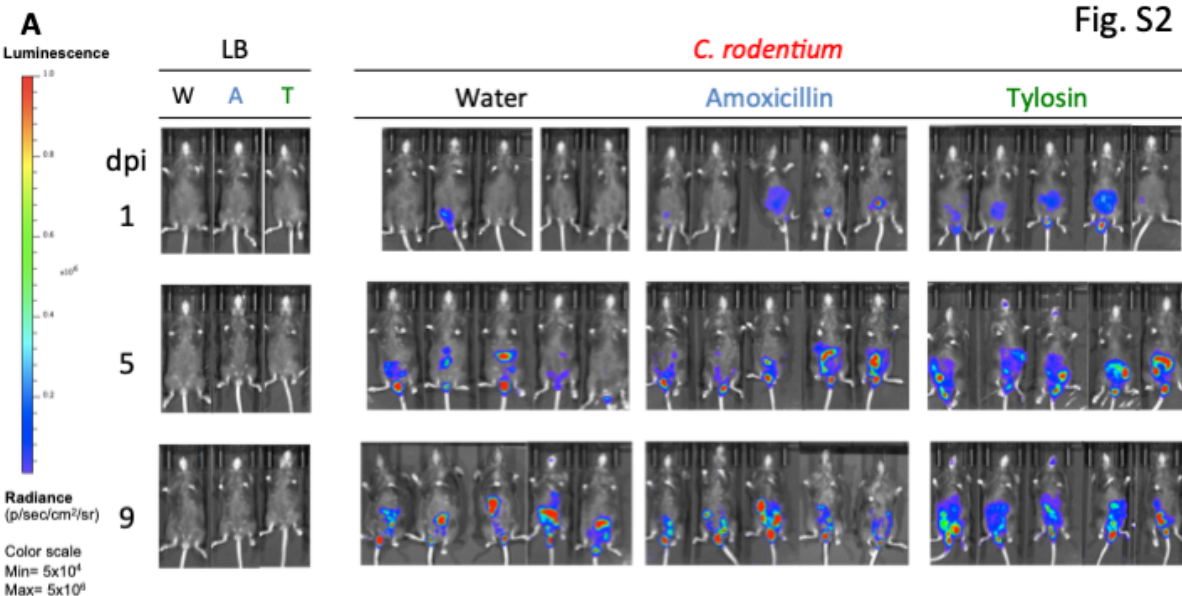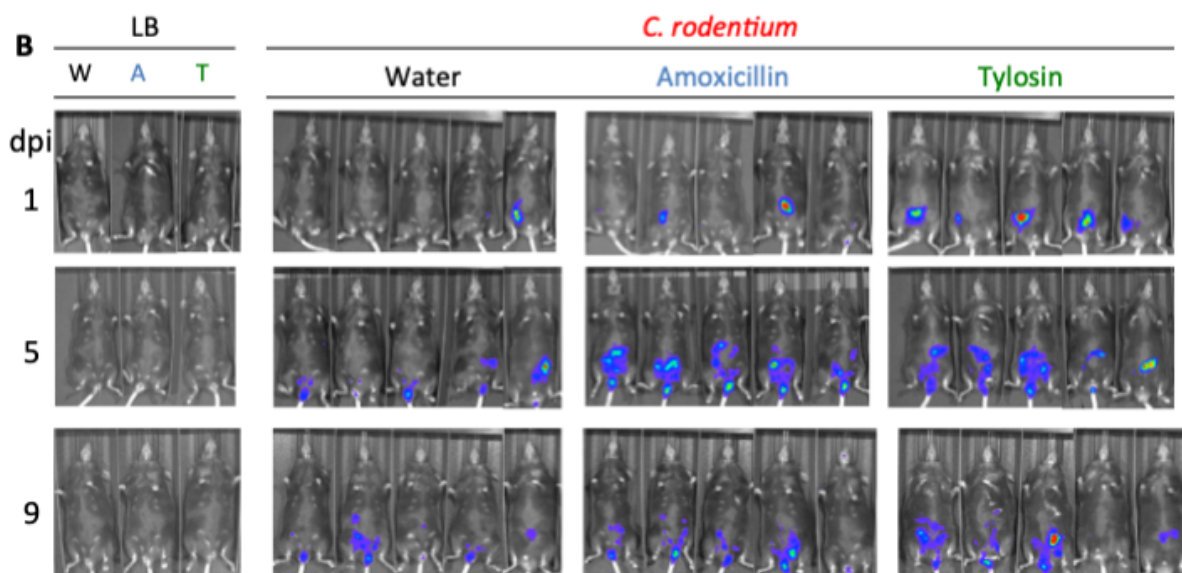

Supplement: FIG S2 [file mBio.02820-19-sf002.pdf]

Fig S3

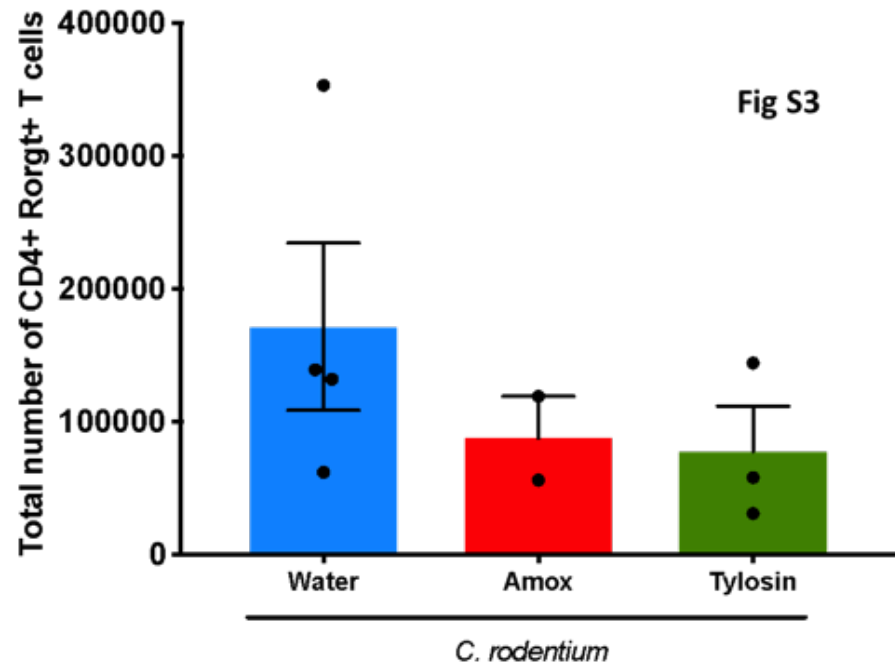

Supplement: FIG S3 [file mBio.02820-19-sf003.pdf]
