# Supplementary material for: Long-Term Effects of Early-Life Antibiotic Exposure on Resistance to Subsequent Bacterial Infection
Source: mBio. 2019 Dec 24;10(6):e02820-19. doi: 10.1128/mBio.02820-19 (PMC6935859; doi:10.1128/mBio.02820-19)
Supplement: TABLE S1 [file mBio.02820-19-st001.docx]

**Supplementary Table 1. Summary of Adonis and Anosim testing of Unweighted UniFrac distances, related to Figure 2, Panel B**

|  | | | p-values from Adonis testing^a^ by days post-infection | | |
| --- | --- | --- | --- | --- | --- |
| Group Comparison | | | –3 | 4 | 11 |
| Tylosin | vs. | Water | 0.001 | n/a | n/a |
| Tylosin+*CR* | vs. | Tylosin+LB | n/a | 0.037 | 0.070 |
| Tylosin+*CR* | vs. | Water+*CR* | n/a | 0.008 | 0.014 |
| Tylosin+*CR* | vs. | Water+LB | n/a | 0.069 | 0.068 |
| Tylosin+LB | vs. | Water+*CR* | n/a | 0.017 | 0.020 |
| Tylosin+LB | vs. | Water+LB | n/a | 0.112 | 0.112 |
| Water+*CR* | vs. | Water+LB | n/a | 0.037 | 0.052 |
|  | | | p-values from Anosim testing^a^ by days post-infection | | |
| Group Comparison | | | –3 | 4 | 11 |
| Tylosin | vs. | Water | 0.001 | n/a | n/a |
| Tylosin+*CR* | vs. | Tylosin+LB | n/a | 0.043 | 0.075 |
| Tylosin+*CR* | vs. | Water+*CR* | n/a | 0.008 | 0.014 |
| Tylosin+*CR* | vs. | Water+LB | n/a | 0.076 | 0.075 |
| Tylosin+LB | vs. | Water+*CR* | n/a | 0.017 | 0.043 |
| Tylosin+LB | vs. | Water+LB | n/a | 0.112 | 0.112 |
| Water+*CR* | vs. | Water+LB | n/a | 0.043 | 0.081 |

^a^FDR-corrected; n/a, not applicable.
