# Supplementary material for: Long-Term Effects of Early-Life Antibiotic Exposure on Resistance to Subsequent Bacterial Infection
Source: mBio. 2019 Dec 24;10(6):e02820-19. doi: 10.1128/mBio.02820-19 (PMC6935859; doi:10.1128/mBio.02820-19)
Supplement: TABLE S2 [file mBio.02820-19-st002.docx]

**Supplementary Table 2. Summary of Adonis and Anosim testing of Unweighted UniFrac distances, related to Figure 4, Panel C**

| Group Comparison | | | p-value from Adonis testing^a^ | p-value from Anosim testing^a^ |
| --- | --- | --- | --- | --- |
| Water | vs. | Amoxicillin | 0.002 | 0.002 |
| Water | vs. | Tylosin | 0.002 | 0.002 |
| Amoxicillin | vs. | Tylosin | 0.002 | 0.002 |

^a^FDR-corrected.
