# Supplementary material for: Long-Term Effects of Early-Life Antibiotic Exposure on Resistance to Subsequent Bacterial Infection
Source: mBio. 2019 Dec 24;10(6):e02820-19. doi: 10.1128/mBio.02820-19 (PMC6935859; doi:10.1128/mBio.02820-19)
Supplement: TABLE S3 [file mBio.02820-19-st003.docx]

**Supplementary Table 3. Summary of Adonis and Anosim testing of Unweighted UniFrac distances, related to Figure 6, Panel A**

|  | | | p-values from Adonis testing^a^ by day of life | | | | | |
| --- | --- | --- | --- | --- | --- | --- | --- | --- |
| Group Comparison | | | 24 | 60 | 88 | 91 | 98 | 101 |
| Water | vs. | Amoxicillin | 0.001 | 0.001 | 0.001 | n/a | n/a | n/a |
| Water | vs. | Tylosin | 0.001 | 0.001 | 0.001 | n/a | n/a | n/a |
| Amoxicillin | vs. | Tylosin | 0.002 | 0.001 | 0.001 | n/a | n/a | n/a |
| Amoxicillin+*CR* | vs. | Amoxicillin+LB | n/a | n/a | n/a | 0.105 | 0.033 | 0.039 |
| Amoxicillin+*CR* | vs. | Tylosin+*CR* | n/a | n/a | n/a | 0.020 | 0.019 | 0.038 |
| Amoxicillin+*CR* | vs. | Tylosin+LB | n/a | n/a | n/a | 0.037 | 0.032 | 0.036 |
| Amoxicillin+*CR* | vs. | Water+*CR* | n/a | n/a | n/a | 0.020 | 0.023 | 0.039 |
| Amoxicillin+LB | vs. | Tylosin+*CR* | n/a | n/a | n/a | 0.026 | 0.023 | 0.031 |
| Amoxicillin+LB | vs. | Tylosin+LB | n/a | n/a | n/a | 0.109 | 0.109 | 0.109 |
| Amoxicillin+LB | vs. | Water+*CR* | n/a | n/a | n/a | 0.029 | 0.025 | 0.029 |
| Tylosin+*CR* | vs. | Tylosin+LB | n/a | n/a | n/a | 0.097 | 0.023 | 0.058 |
| Tylosin+*CR* | vs. | Water+*CR* | n/a | n/a | n/a | 0.020 | 0.016 | 0.019 |
| Tylosin+LB | vs. | Water+*CR* | n/a | n/a | n/a | 0.029 | 0.029 | 0.025 |
|  | | | p-values from Anosim testing^a^ by day of life | | | | | |
| Group Comparison | | | 24 | 60 | 88 | 91 | 98 | 101 |
| Water | vs. | Amoxicillin | 0.001 | 0.001 | 0.001 | n/a | n/a | n/a |
| Water | vs. | Tylosin | 0.001 | 0.001 | 0.001 | n/a | n/a | n/a |
| Amoxicillin | vs. | Tylosin | 0.002 | 0.001 | 0.001 | n/a | n/a | n/a |
| Amoxicillin+*CR* | vs. | Amoxicillin+LB | n/a | n/a | n/a | 0.442 | 0.066 | 0.053 |
| Amoxicillin+*CR* | vs. | Tylosin+*CR* | n/a | n/a | n/a | 0.024 | 0.041 | 0.167 |
| Amoxicillin+*CR* | vs. | Tylosin+LB | n/a | n/a | n/a | 0.051 | 0.047 | 0.050 |
| Amoxicillin+*CR* | vs. | Water+*CR* | n/a | n/a | n/a | 0.024 | 0.040 | 0.128 |
| Amoxicillin+LB | vs. | Tylosin+*CR* | n/a | n/a | n/a | 0.092 | 0.030 | 0.128 |
| Amoxicillin+LB | vs. | Tylosin+LB | n/a | n/a | n/a | 0.125 | 0.128 | 0.128 |
| Amoxicillin+LB | vs. | Water+*CR* | n/a | n/a | n/a | 0.097 | 0.116 | 0.043 |
| Tylosin+*CR* | vs. | Tylosin+LB | n/a | n/a | n/a | 0.400 | 0.066 | 0.146 |
| Tylosin+*CR* | vs. | Water+*CR* | n/a | n/a | n/a | 0.024 | 0.028 | 0.025 |
| Tylosin+LB | vs. | Water+*CR* | n/a | n/a | n/a | 0.066 | 0.131 | 0.034 |

^a^FDR-corrected; n/a, not applicable.
